# From Western Asia to the Mediterranean Basin: Diversification of the Widespread *Euphorbia nicaeensis* Alliance (Euphorbiaceae)

**DOI:** 10.3389/fpls.2022.815379

**Published:** 2022-06-23

**Authors:** Valentina Stojilkovič, Eliška Záveská, Božo Frajman

**Affiliations:** ^1^Department of Botany, University of Innsbruck, Innsbruck, Austria; ^2^Department of Biology, Biotechnical Faculty, University of Ljubljana, Ljubljana, Slovenia; ^3^Institute of Botany of the Czech Academy of Sciences, Průhonice, Czechia

**Keywords:** Eurasian steppes, Mediterranean Basin, Irano-Turanian region, morphometry, phylogeny, polyploidy, RAD sequencing, taxonomy

## Abstract

The Mediterranean Basin is an important biodiversity hotspot and one of the richest areas in the world in terms of plant diversity. Its flora parallels in several aspects that of the Eurasian steppes and the adjacent Irano-Turanian floristic region. The *Euphorbia nicaeensis* alliance spans this immense area from the western Mediterranean to Central Asia. Using an array of complementary methods, ranging from phylogenomic and phylogenetic data through relative genome size (RGS) estimation to morphometry, we explored relationships and biogeographic connections among taxa of this group. We identified the main evolutionary lineages, which mostly correspond to described taxa. However, despite the use of highly resolving Restriction Site Associated DNA (RAD) sequencing data, relationships among the main lineages remain ambiguous. This is likely due to hybridisation, lineage sorting triggered by rapid range expansion, and polyploidisation. The phylogenomic data identified cryptic diversity in the Mediterranean, which is also correlated with RGS and, partly, also, morphological divergence, rendering the description of a new species necessary. Biogeographic analyses suggest that Western Asia is the source area for the colonisation of the Mediterranean by this plant group and highlight the important contribution of the Irano-Turanian region to the high diversity in the Mediterranean Basin. The diversification of the *E. nicaeensis* alliance in the Mediterranean was triggered by vicariance in isolated Pleistocene refugia, morphological adaptation to divergent ecological conditions, and, to a lesser extent, by polyploidisation.

## Introduction

The Mediterranean Basin is an important biodiversity hotspot and one of the richest areas in the world in terms of animal and plant diversity (Myers et al., 2000), harbouring 24,000 plant species, of which 60% are endemic (Greuter, 1991). Its rich biota is a result of a complex palaeogeologic and paleoclimatic history as well as current ecogeographical heterogeneity (Blondel and Aronson, 1999; Thompson, 2005; Blondel et al., 2010; Nieto Feliner, 2014). Because of its geological and climatic complexity, it provides an ideal area for studying biogeography and evolution (Ståhls et al., 2016). The western Mediterranean is geologically older (Krijgsman, 2002); however, the eastern Mediterranean is considered to be more diverse (Nieto Feliner, 2014), thus acting as a reservoir for evolution of plants and as a cradle for the diversification of different lineages, which is, in particular, true for the Balkan Peninsula (Mansion et al., 2009; Roquet et al., 2009). Despite a high amount of studies dedicated to Mediterranean biodiversity, there are still gaps in our understanding how the Basin has come to be one of Earth’s biodiversity hotspots (Nieto Feliner, 2014).

The Irano-Turanian (IT) floristic region, easterly adjacent to the Mediterranean, is another important hotspot of biodiversity, representing the meeting point of western and eastern floras of the Holarctic Kingdom, with many Mediterranean lineages having originated in the IT region (Zohary, 1973; Quézel, 1985; Manafzadeh et al., 2014, 2017). The western IT region is floristically richer than the eastern one, harbouring approximately 27,000 plant species, many of which are endemic (Takhtajan, 1986; Manafzadeh et al., 2017). Especially central Anatolia, a transition zone between Mediterranean and IT floras (Davis, 1971; Takhtajan, 1986), is characterised by high species endemism of presumably relatively recent origin (Davis and Hedge, 1975; Noroozi et al., 2019). In the same line, the easterly adjacent Armenian and Iranian plateaus, known for their heterogeneous flora rich in endemic genera and species (Hedge and Wendelbo, 1978), belong to the most active centres of speciation in the IT region (Boissier, 1867; Takhtajan, 1986; Mahmoudi Shamsabad et al., 2021).

North-easterly adjacent to the Eastern Mediterranean and western parts of the IT region, the second-largest continuous biome on Earth, the Eurasian steppes of the Circumboreal floristic region (Takhtajan, 1986) are spanning from Central and Eastern Europe (Pannonian and Pontic areas) to Central Asia (Lal, 2004; Wesche et al., 2016; Kirschner et al., 2020), where they extend to the IT region. They represent a large fraction of temperate grasslands (Coupland, 1993; Lavrenko and Karamysheva, 1993) and play an important ecological role, representing a complex array of microniches (Wilson et al., 2012) and serving as a global carbon sink (Lal, 2004). These grasslands, which are shaped by strongly seasonal climates (Peart, 2008), share several characteristics with the Mediterranean grasslands, which is reflected in the sharing of multiple plant genera and species across both biomes (Hamasha et al., 2012). In a recent study, Kirschner et al. (2020) have shown that there is a strong phylogeographic break between the azonal European steppes (roughly east of the Carpathians) and the zonal East European-Asian steppes in a number of plant and animal species.

One of the plant groups having the highest diversity in mountainous areas of the Mediterranean Basin and in the IT region, with some species inhabiting the steppes of Eurasia, is *Euphorbia* L. sect. *Pithyusa* (Raf.) Lázaro. With 50 species, this is one of the larger sections of *Euphorbia* subgen. *Esula* Pers. (Riina et al., 2013). Within this section, there is a lineage comprising c. 15 species distributed from Morocco and the Iberian Peninsula in the west to Central Asia in the east and referred to as the *E. barrelieri-nicaeensis-seguieriana* clade by Frajman et al. (2019). The onset of the diversification of this clade, with largely unresolved interspecific relationships, was dated as Pleistocene (Horn et al., 2014; Frajman and Schönswetter, 2017). Whereas Frajman and Schönswetter (2017) and Frajman et al. (2019) explored evolutionary relationships within the *E. barrelieri* Savi and *E. seguieriana* Neck. alliances, little is known about species delimitations and phylogenetic relationships within the *E. nicaeensis* alliance. *Euphorbia nicaeensis* All., described from Nice (France), is according to most recent taxonomic treatments (e.g., Flora Europaea, Radcliffe-Smith and Tutin, 1968; Med-Check list, Greuter et al., 1986) a morphologically variable and widespread species, distributed from Morocco and Iberia in the west to Anatolia and western Russia in the east. Radcliffe-Smith and Tutin (1968) claimed that “a number of, more or less, local populations can be recognised and have often been given a specific rank, but the distinctions between them are of a minor nature and do not seem to be clear-cut, but two subspecies can be recognised,” namely southern European (Mediterranean) *E. nicaeensis* subsp. *nicaeensis* and eastern and central European *E. nicaeensis* subsp. *glareosa* (Pall. ex M. Bieb.). In contrast, Greuter et al. (1986) recognised six additional subspecies alongside subsp. *glareosa* and subsp. *nicaeensis*: two from Italy [*E. nicaeensis* subsp. *japygica* (Ten.) Arcangeli and subsp. *prostrata* (Fiori) Arrigoni] and four from Eastern Europe [*E. n.* subsp. *cadrilateri* (Prodan) Kuzmanov, subsp. *dobrogensis* (Prodan) Kuzmanov, subsp. *goldei* (Proh.) Greuter and Burdet and subsp. *stepposa* (Zoz) Greuter and Burdet]. Such a treatment with a broadly circumscribed *E. nicaeensis*, including a varying number of subspecies, was also implemented in most national floras and taxonomic treatments (e.g., Kuzmanov, 1979; Radcliffe-Smith, 1982; Micevski, 1998; Govaerts et al., 2000). In contrast, some, mostly earlier, authors separated the Mediterranean *E. nicaeensis* from the central/eastern European species, and different names have been applied for the latter, e.g., *E. glareosa* Pall. ex M. Bieb. (Beck-Mannagetta, 1920; Janković and Nikolić, 1972) or *E. pannonica* Host. (Hegi, 1966). Some authors (e.g., Prokhanov, 1949; Geltman, 2009, 2020) recognised even several species that were in the above-mentioned treatments mostly considered synonyms or subspecies: *E. glareosa*, *E. goldei* Prokh., *E. stepposa* Zoz, and *E. volgensis* Krysth. Some additional species, e.g., *E. erythrodon* Boiss. and Heldr. and *E. petrophila* C. A. Mey. from the Pontic and IT regions, were also included in the *E. nicaeensis* alliance by Radcliffe-Smith (1982) and Geltman (2020).

Phylogenetic studies of Internal Transcribed Spacer (ITS) sequences, including a limited number of samples, resolved the Mediterranean *E. nicaeensis* separated from the eastern European and Asian populations treated under *E. glareosa*, *E. pannonica*, *E. petrophila*, and *E. stepposa*. These species either formed a grade of accessions successively sister to the *E. nicaeensis* clade, or a poorly supported sister clade to the *E. nicaeensis* clade, which also included *E. hercegovina* Beck (Frajman and Schönswetter, 2011, 2017; Riina et al., 2013; Frajman et al., 2019). *Euphorbia hercegovina* is endemic to the western Balkan Peninsula between Montenegro and Bosnia and Herzegovina and was, in the past, considered closely related or conspecific with *E. barrelieri* Savi (e.g., Poldini, 1969; Greuter et al., 1986; Govaerts et al., 2000; Trinajstić, 2007; Geltman, 2009); however, Frajman and Schönswetter (2017) showed that it does not belong to the *E. barrelieri* group. In the most recent study by Frajman et al. (2019), *E. macroclada* Boiss. (one individual sampled) was included in the same clade as *E. hercegovina* and *E. nicaeensis* in the nuclear dataset; plastid sequences, however, positioned *E. macroclada* close to the western Asian *E. cheiradenia* Boiss. (Riina et al., 2013).

*Euphorbia macroclada* is morphologically similar to *E. nicaeensis* and was also included in *E.* ser. *Nicaeenses* Prokh. by Prokhanov (1949). It is distributed predominately in Anatolia (Turkey) but extends its range to adjacent Syria, Iraq, Iran, and Armenia (Radcliffe-Smith, 1982). In a Restriction Site Associated DNA Sequencing (RAD) phylogeny based on limited sampling (Frajman et al., 2019), one population of *E. macroclada* was inferred as sister to three populations of *E. nicaeensis* (*E. hercegovina* was not included in the study), and one population of *E. glareosa* was sister to them. Thus, relationships between and within the *E. nicaeensis* and *E. glareosa* lineages still remain unresolved, and it is not clear how the evolutionary relationships are reflected in morphological differentiation and species delimitations or in their spatial distribution. In addition, it remains unknown what role the Mediterranean Basin, the IT region, and the Eurasian steppes of the Circumboreal region played in diversification of this widespread group. Finally, even if polyploidy seems to be rare in *E.* subgen. *Esula* (Frajman and Schönswetter, 2011; Riina et al., 2013), recent studies, including a dense geographic sampling, have revealed polyploidisation events in several groups of *Euphorbia* (e.g., Stevanoski et al., 2020; Caković et al., 2021). The diploid chromosome number (2*n* = 18) appears to be the most common within the *E. nicaeensis* alliance, but there is also a report of 2*n* = 56 for *E. nicaeensis* in Spain and a recent report of a tetraploid (2*n* = 36) population of *E. macroclada* from Turkey (Rice et al., 2015; Genç and Kültür, 2020), indicating that polyploidisation might be one of the processes driving diversification within our study group.

The aim of our study is to disentangle phylogenetic relationships within the *E. nicaeensis* alliance and to determine its position within the *E. barrelieri-nicaeensis-seguieriana* clade. After identifying clear evolutionary lineages using next-generation RADseq as well as nuclear ribosomal ITS sequences, we explored biogeographic relationships within the *E. nicaeensis* alliance, specifically amongst *E. hercegovina*, *E. macroclada*, and *E. nicaeensis* (hereafter, the *E. nicaeensis* lineage) and their relation to *E. erythrodon*, *E. glareosa* s.l. (including *E. pannonica* and *E. stepposa*), and *E. petrophila* (hereafter, the *E. glareosa* lineage). We explored which geographic region served as a source for the Mediterranean taxa and how their diversity is distributed within this biome. Given the highest taxonomic diversity in the western IT region, we hypothesise that this region was the source for the Mediterranean taxa of the *E. nicaeensis* alliance, as evidenced in many other groups (Manafzadeh et al., 2017), and that Pleistocene glaciations had an important role in driving diversification of this lineage, in line with previous evidence (Nieto Feliner, 2014; Caković et al., 2021). This was the case also within the *E. verrucosa* L. group (Caković et al., 2021), which strongly overlaps with *E. nicaeensis*, both in distribution and habitats. In addition, we intersected the phylogenetic data with relative genome size (RGS) data and assessed the incidence of polyploidisation within our study group. Finally, by also intersecting morphometric data with phylogenetic patterns, we are able to discuss reasons for observed incongruences. By identifying cryptic diversity within the Mediterranean, we propose a revised taxonomic treatment, including the description of a new species and revised distribution data for the studied taxa.

## Materials and Methods

### Plant Material

**FIGURE 1:**
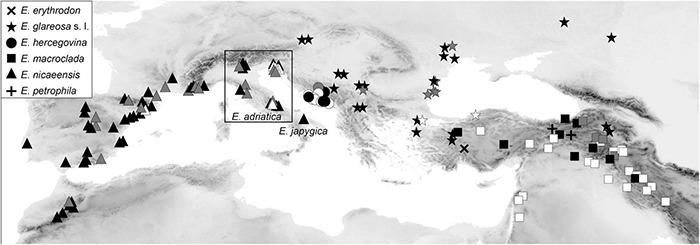
Distribution of *Euphorbia nicaeensis* alliance populations sampled and used in this study. Black symbols indicate populations used in genetic (RADseq and/or ITS) and, mostly, also, in relative genome size (RGS) and morphometric analyses. Grey symbols indicate additional populations used in RGS and/or morphometric analyses; white symbols indicate populations used only in morphometric analyses. The corresponding population numbers are presented in Supplementary Figure 1, and details are given in Supplementary Table 1. *Euphorbia adriatica* and *E. japygica* have been previously included in *E. nicaeensis* but are, based on our results, independent species.

Plant material for molecular analyses and RGS estimation (silica-gel dried leaf material), as well as for morphometric analyses (herbarium vouchers), was collected in the field between 2006 and 2019. We additionally sequenced ITS from 18 herbarium specimens from the herbaria MA (16), WU (1) and the private herbarium of W. Gutermann (1); a herbarium specimen of the outgroup *E. humilis* Ledeb. from FRU was used for RADseq. In addition, 32 herbarium specimens from herbaria M (10) and W (22) were used for morphometric analyses. In total, 145 populations of *E. erythrodon* (one population), *E. glareosa* s.l. (29 populations), *E. hercegovina* (13 populations), *E. macroclada* (34 populations), *E. nicaeensis* (66 populations; including population 58 of *E. nicaeensis* subsp. *japygica*, which we name *E. japygica* hereafter for simplicity), and *E. petrophila* (2 populations), as well as 30 populations of 14 outgroup taxa were studied (Figure 1, Supplementary Figure 1, and Supplementary Table 1). For the focal taxa *E. hercegovina*, *E. macroclada*, and *E. nicaeensis*, our sampling covers their complete distributions. In the case of *E. hercegovina* and *E. nicaeensis*, material from areas corresponding to the type localities was included in most analyses, including RADseq (populations 77 and 40, respectively), whereas, in the case of *E. macroclada*, the population 81 is ca. 250 km away from the type locality. In the case of *E. glareosa* s.l., which includes different taxa as outlined in the introduction, we studied several samples likely belonging to different taxa (species or subspecies), but it was beyond the aims of this study to include type populations of all these taxa. The same was the case for *E. erythrodon* and *E. petrophila*.

### Restriction Site Associated DNA Sequencing Library Preparation

Total genomic DNA was extracted from dried leaf tissue (ca. 10 mg) using the CTAB protocol described by Frajman and Schönswetter (2011) and purified with the NucleoSpin gDNA clean-Up kit (Macherey-Nagel, Düren, Germany). Single-digest RAD libraries were prepared using the restriction enzyme PstI (New England Biolabs, Ipswich, MA, United States) and a protocol adopted from Paun et al. (2015). Briefly, we started with 110 ng DNA per individual and ligated 100 mM P1 adapters to the restricted samples overnight at 16°C. Shearing by sonication was performed with an M220 Focused-ultrasonicator (Covaris) with settings targeting a size range of 200 to 800 bp and peak at 400 bp. A total of 115 individuals (65 individuals from 32 ingroup populations and 52 individuals from 26 populations of various outgroup taxa from *E.* sect. *Pithyusa*; see Supplementary Table 1 for details) were sequenced on an Illumina HiSeq 2500 at CSF Vienna^1^ as 100 bp single reads using Illumina chemistry.

### Identification of Restriction Site Associated DNA Sequencing Loci and Single Nucleotide Polymorphism Calling

The raw reads were quality filtered and demultiplexed according to individual barcodes using the Picard command line tool BamIndexDecoder^2^ and the program “process_radtags.pl” implemented in Stacks v2.3d (Catchen et al., 2011, 2013). The RAD loci were further assembled, and Single Nucleotide Polymorphism (SNPs) were called using the “denovo_map.pl” pipeline in Stacks. A dataset used for subsequent phylogenetic reconstruction was built using a minimum × 5 coverage to identify a stack (-m 5), a maximum number of five differences between two stacks per locus per sample (-M 5), and a maximum number of five differences among loci to be considered as orthologous across multiple samples (-n 5). The parameter values (-m, -M, -n) were previously optimised for *E. seguieriana* and other species, here used as the outgroup, as described in Kirschner et al. (2020) and similarly applied by Frajman et al. (2019). The program “populations” in the Stacks package was used: (i) to identify samples with missing data > 28%, to exclude them from further analyses; (ii) to pre-filter loci by setting the maximum observed heterozygosity to 0.65 to process a nucleotide site at a locus (and prevent processing of paralogs); and (iii) to export an SNP dataset in vcf format. Starting with a vcf file, we further filtered out sites with depth of coverage < × 10 and sites with > 50% missing samples, using VCFtools v0.1.15 (Danecek et al., 2011). The filtered vcf file was converted to phylip format using the python script vcf2phylip (Ortiz, 2019) and used for downstream phylogenetic reconstructions.

### Phylogenetic Analyses, Species Tree Inference, and Ancestral Area Estimation Based on the Complete RADseq Dataset

To infer phylogenetic relationships among all 115 individuals, we computed a maximum likelihood (ML) phylogeny using RAxML v8.2.8 (Stamatakis, 2014). Invariant sites were removed from the original phylip format using the script “deleteAlignColumn.pl”^3^, and Felsenstein’s ascertainment bias correction was further used to account for missing invariant sites as recommended by Leaché et al. (2015). Tree searches were done under a GTR model with categorical optimisation of substitution rates (ASC_GTRCAT; Stamatakis, 2014). *Euphorbia cheiradenia*, *E. kopetdaghi* (Prokh.) Prokh., *E. matritensis* Boiss., *E. minuta* Loscos and Pardo, and *E. polycaula* Boiss. and Hohen. were used for rooting based on Riina et al. (2013) and their placement in a preliminary NeighborNet constructed using SplitsTree4 v12.3 (Huson and Bryant, 2006). The best-scoring ML tree was bootstrapped using the frequency-based stopping criterion (Pattengale et al., 2010).

To infer a species tree and estimate divergence times, we applied SNAPP v1.5.1 (Bryant et al., 2012) implemented in BEAST v2.6.4 (Bouckaert et al., 2014) as described by Stange et al. (2018). We used a reduced data set containing 48 individuals that represented the 19 main lineages identified by RAxML, mainly corresponding to species; *E. nicaeensis* excepted, for which two lineages were resolved and treated independently in species tree inference. Two populations, corresponding to *E. erythrodon* 80 and *E. glareosa* 125, which Structure analyses showed to be introgressed (see section “Results”), were excluded from species tree inference. We constructed a new RAD data subset for these 19 lineages using the filtering parameters described above, but requiring loci to be shared among all 19 lineages. We randomly selected one SNP per locus and further filtered for biallelic SNPs only, achieving 1,498 SNPs in total. To scale the tree, we used a secondary calibration by setting the prior age of the root to 4.25 Mya with a normally distributed SD of 1.7, which corresponds to the median age and 95% highest posterior density (HPD) interval of the split between *E. kopetdaghii* and *E. nicaeensis* in Horn et al. (2014). To improve mixing and convergence of the model, we constrained the monophyly of both ingroup and outgroup, as they were consistently resolved as monophyletic with strong support in our RAxML analysis. We ran two independent MCMCs for 100,000 generations, discarding 30% of the generations as burn-in. Log files were analysed using TRACER v1.6 to assess convergence and ensure that the effective sample size (ESS) for all parameters was > 200 (Rambaut et al., 2014). We estimated the maximum clade credibility (MCC) tree from the posterior distributions of both runs using TreeAnnotator v2.5.0 (Drummond and Rambaut, 2007).

**FIGURE 2:**
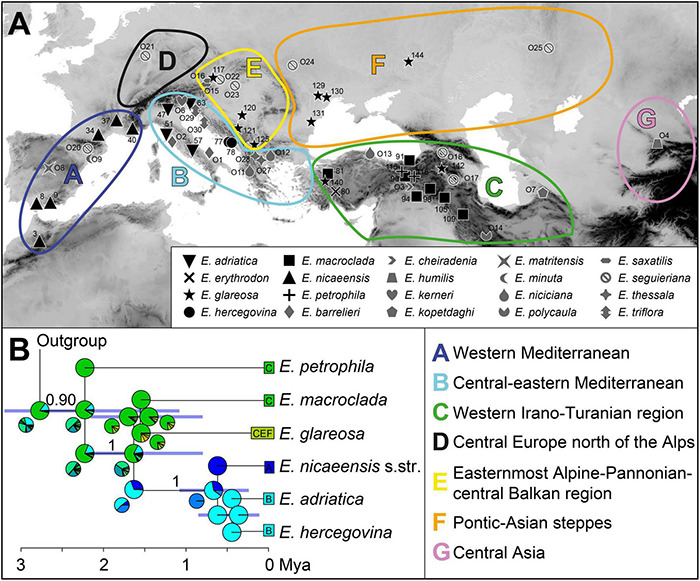
**(A)** Geographic areas and geographic provenance of the investigated populations. **(B)** The time-calibrated species tree inferred with SNAPP and used in the biogeographic analysis with BioGeoBEARS. Numbers above branches are posterior probabilities > 0.80, and the horizontal bars correspond to 95% highest posterior densities (HPD) for the age estimates. Pie charts at each node show the marginal probabilities of alternative ancestral ranges obtained from the BioGeoBEARS analysis under the DEC + J model. In addition, smaller pie charts resulting from the DEC model are presented in cases where the reconstruction between the models differed. Colours in pie charts correspond to the geographic areas in A. The trees, including outgroup taxa, are presented in Supplementary Figure 3. Population numbers correspond to Supplementary Table 1.

We performed a biogeographic analysis with BioGeoBEARS (Matzke, 2013), using the MCC tree inferred with SNAPP, and defined seven geographic areas (Figure 2A). We combined the knowledge accrued on floristic patterns to designate geographic areas [e.g., that of Takhtajan (1986), largely adopted by Manafzadeh et al. (2017), for the IT region], while also considering more recent phylogeographic studies (e.g., Kirschner et al., 2020). The boundaries of the areas were also adapted to our phylogenetic results, i.e., the borders were drawn in the areas for which we identified main phylogenetic breaks. The Mediterranean region *sensu* Takhtajan (1986) was split into western Mediterranean (A) and central-eastern Mediterranean (B), following the main split within *E. nicaeensis* and distribution patterns of some co-occuring western Mediterranean species outlined by Caković et al. (2021). The third area corresponds to the western IT floristic region (C) *sensu* Manafzadeh et al. (2017), including Anatolia, the Caucasus, and the Armenian and Iranian plateaus, which largely corresponds to the distribution of *E. macroclada*, but also to that of *E. petrophila* and *E. erythrodon*. It remains unclear whether the populations of *E. petrophila* from Krym (Area F) and Lesbos, in the Aegean area (Area B), are in the same lineage as Anatolian *E. petrophila*; therefore, we mapped this species only to the area C. We also included the *E. niciciana* O13 population south of the Black Sea, in Region C, as this species is mainly found in Anatolia and the here sampled population grouped with other samples from Anatolia in the study by Frajman et al. (2019). Region D (central Europe north of the Alps) included only a single population of *E. seguieriana*, which was in a clearly divergent clade in the study of Frajman et al. (2019). In contrast, the samples from the easternmost Alpine-Pannonian-central Balkan area were included in its own region E. The latter region includes the azonal steppes of Takhtajan’s (1986) Circumboreal floristic region, since they were clearly divergent from the Pontic-Asian steppes (hereafter, Area F), for a number of plant and animal taxa, in Kirschner et al. (2020). The final area, central Asia (G), includes a single population of *E. humilis*, species that is isolated from all other members of the *E. barrelieri-nicaeensis-seguieriana* clade. We used six biogeographic models in a common likelihood framework: a likelihood version of Dispersal-Vicariance analysis (DIVALIKE; Ronquist, 1997), LAGRANGE Dispersal and Extinction Cladogenesis (DEC model, Ree et al., 2005; Ree and Smith, 2008), a likelihood version of BayArea (Landis et al., 2013), and an alternative version for each of the models that include founder-event speciation (+J). It was, however, claimed by Ree and Sanmartin (2018) that statistical comparisons of likelihoods between DEC and DEC+J are inappropriate (but see Matzke, 2013^4^). The maximum number of areas for each node was set to four, which is the maximum number of areas occupied by the extant taxa (Ronquist, 1996; Hilpold et al., 2014). Each terminal node in the tree was coded with the total distribution area of the taxon/lineage. We defined a dispersal probability matrix to determine the effect of geographic distance on dispersal ability. The rate of dispersal between adjacent areas was set to 1 (e.g., between the western and the eastern Mediterranean), between non-adjacent but geographically close areas to 0.75 (e.g., between the western Mediterranean and northern Europe), between more distant areas to 0.5 (e.g., between the western Mediterranean and Anatolia), and between the most distant areas to 0.25 (e.g., between the western Mediterranean and Central Asia), following Hilpold et al. (2014). After running the six models, we compared the results with a likelihood ratio test, applying the Akaike Information Criterion to select the best fit model.

### Genetic Structure Within the *Euphorbia nicaeensis* Alliance Based on Restriction Site Associated DNA Sequencing Data

To explore in more detail the phylogenetic relationships and potential presence of gene flow within the *E. nicaeensis* alliance, we analysed a subset of nine main lineages, inferred from the complete dataset with RAxML, by using Bayesian clustering and SNAPP. The nine lineages corresponded to the monophyletic species *E. erythrodon*, *E. hercegovina*, and *E. macroclada*, and, for *E. glareosa*, *E. nicaeensis*, and *E. petrophila*, which appeared poly- or paraphyletic in the RAxML tree, two lineages were delimited in each case. This additional analysis was performed because, in the analysis of the complete dataset, where several distantly related lineages (e.g., that of *E. polycaula*) were included, the dataset included only 1,498 SNPs due to larger amounts of missing data across different lineages and filtering for no missing data needed for SNAPP analysis. When analysing only closely related species of the *E. nicaeensis* alliance, and including one less-distant outgroup (*E. triflora*), the amount of SNPs after filtering was much higher, and 3,000 unlinked SNPs were then used for the SNAPP analysis, resulting in better resolved relationships (i.e., better support in terms of PP values).

For the Bayesian clustering analysis, a set of RADseq loci was exported into Structure format using the –structure flag, while sampling loci shared by at least 10% of populations and 40% of individuals within those populations (using -p and -r flags). One SNP per locus (–write-single-snp flag) was selected to minimise the chance of selecting linked loci, resulting in 15,978 SNPs in total. For 65 individuals representing nine lineages of the *E. nicaeensis* alliance, the optimal grouping of populations was determined using fastSTRUCTURE v1.0 (Raj et al., 2014). The analysis was performed for K (number of groups), ranging from 1 to 10, with the script *structure.py*, using a simple prior. The optimal number of K was determined using the script *chooseK.py*; both scripts are part of the fastSTRUCTURE package.

The same dataset, but including *E. triflora* as the outgroup and further filtered for absence of missing data, was used as input for SNAPP analysis, resulting in 3,000 unlinked SNPs. The analysis was performed as described above for the entire dataset, but applying to the split between *E. triflora* and the ingroup a secondary calibration, set to 3.3 Mya with a normally distributed SD of 0.9, derived from estimating divergence times for the entire dataset. A topological constraint was applied to the monophyletic groups inferred by RAxML analysis for the entire dataset as outlined above.

### Internal Transcribed Spacer Sequencing and Analyses of Sequence Data

Internal Transcribed Spacer sequencing, contig assembly and editing, and sequence alignment were performed as described by Frajman and Schönswetter (2011), with modifications described by Cresti et al. (2019). We sequenced ITS for one individual per population from 58 ingroup populations and included 12 ingroup and seven outgroup GenBank sequences selected based on Riina et al. (2013) and Frajman et al. (2019) (Supplementary Table 1). The final ITS alignment thus consisted of one sequence of *E. erythrodon*, 17 of *E. glareosa* s.l., six of *E. hercegovina*, one of *E. japygica*, ten of *E. macroclada*, 34 of *E. nicaeensis*, and one of *E. petrophila*, resulting in a much larger dataset with denser geographic sampling of the focal taxa, compared to the RADseq analysis. GenBank numbers are given in Supplementary Table 1.

Maximum parsimony (MP) and MP bootstrap (MPB) analyses were performed using PAUP v4.0b10 (Swofford, 2002) as described by Frajman et al. (2019). Bayesian analyses were performed using MrBayes 3.2.1 (Ronquist et al., 2012), applying the HKY+Γ substitution model proposed by the Akaike information criterion (AIC) implemented in MrAIC.pl v1.4 (Nylander, 2004) and the settings as in Frajman et al. (2019). The posterior probabilities (PP) of the phylogeny and its branches were determined from the combined set of trees, discarding the first 1,001 trees of each run as burn-in. TRACER v1.6 was used to assess convergence and ensure that the ESS for all parameters was > 200 (Rambaut et al., 2014). In addition, a NeighborNet was produced with ITS sequences of *E. erythrodon*, *E. glareosa* s.l., *E. hercegovina*, *E. japygica*, *E. macroclada*, and *E. nicaeensis*, which formed a poorly supported clade in the ITS tree, using SplitsTreev4.12.3 (Huson and Bryant, 2006).

### Relative Genome Size Estimation

Relative genome size was measured using flow cytometry (FCM) as described by Suda and Trávníček (2006). Nuclei of silica gel dried material of *E. erythrodon* (one population), *E. glareosa* s.l. (19 populations), *E. hercegovina* (four populations), *E. japygica* (one population), *E. macroclada* (11 populations), *E. nicaeensis* (38 populations), and *E. petrophila* (two populations), as well as fresh leaves of a reference standard (*Bellis perennis* L., 2C = 3.38 pg; Schönswetter et al., 2007), were stained using 4’,6-diamidino-2-phenylindole (DAPI). If the peaks of the reference standard and the sample overlapped, *Pisum sativum* cv. Kleine Rheinländerin (2C = 8.84 pg; Greilhuber and Ebert, 1994) was used instead. The RGS was estimated for one to five individuals per population (see Supplementary Table 1). A CyFlow space flow cytometer (Partec, GmbH, Münster, Germany) was used to record the relative fluorescence of 3,000 nuclei and FloMax software (Partec) to evaluate histograms and to calculate coefficients of variation (CVs) of both the standard and the sample peaks. The RGS was calculated as the ratio between the values of the mean relative fluorescence of the sample and the standard. For statistical analyses of RGS data, RStudio 1.2.5019 (RStudio Team, 2019, version R-3.6.1), with the visualisation package “ggplot2,” was used. Scatter and box plots were produced for individual samples as well as for species and ploidy levels.

### Morphometric Analyses

We performed morphometric analyses of 16 individuals of *E. glareosa* s.l., 20 *of E. hercegovina*, 32 of *E. macroclada*, and 45 individuals of *E. nicaeensis*. As we were interested in the differentiation among the species and not in the inter-population variability, we analysed one individual per population. The exception was narrowly distributed *E. hercegovina*, for which three to five individuals per population from three populations were analysed (Supplementary Table 1). Our sampling of the two other focal taxa, *E. macroclada* and *E. nicaeensis*, roughly corresponds to their respective range’s size, whereas we only included a limited number of specimens of *E. glareosa* s.l. for comparison.

After initial inspection of 44 morphological characters, we measured or scored 33 characters that showed variation in the investigated taxa and calculated 15 ratios (Table 1). Stem characters were measured manually, whereas the leaf characters were measured partly on scanned herbarium images using ImageJ (Abràmoff et al., 2004) or manually on actual herbarium specimens. All other characters (cyathium, fruit, and seed characters) were measured on images taken with a stereomicroscope Olympus SZX9 using the Olympus image analysis software analySIS pro. Fruits and seeds were developed only in a limited number of specimens. For *E. glareosa* s.l., we measured eleven fruits and nine seeds; for *E. macroclada*, 17 fruits and ten seeds; for *E. nicaeensis*, 18 fruits and 15 seeds, all from different populations, whereas, for *E. hercegovina*, we measured four fruits and four seeds from three populations. In addition, since not all characters were scorable in all individuals, we replaced the missing values in the final data matrix with the species’ mean or mode, the latter in the following characters: number of terminal rays, number of branching of (the longest) terminal ray, and number of fertile axillary rays. Statistical analyses were performed using SPSS 24.0. Correlation among metric characters was tested employing Pearson and Spearman correlation coefficients, and one character from each character pair, yielding a correlation coefficient > 0.90, was excluded from further analyses. Box plot diagrams were produced for all characters in order to visualise and show the variation among four species. After standardisation to zero mean and one unit variance, principal component analysis (PCA) was performed. Subsequently, discriminant analysis (DA) was performed. The PCA and DA analyses were performed separately for (1) vegetative parts of the plants and cyathium characters, for (2) fruit, as well as (3) seed characters, in two steps: for *E. glareosa* s.l., *E. hercegovina*, *E. macroclada*, and *E. nicaeensis* and for closely related *E. hercegovina* and two phylogenetic lineages of *E. nicaeensis* (see section “Results”). Based on the morphometric data, we produced species descriptions and an identification key. Metric values presented there correspond to the 10th and 90th percentiles, supplemented by extreme values in parentheses.

**TABLE 1:** Characters studied in the morphometric analyses of *Euphorbia glareosa, E. hercegovina, E. macroclada*, and *E. nicaeensis.*

No.	Stem
1	Stem length, cm
2	Stem width, cm
3	Stem glabrous/pubescent
	**Pleiochasium**
4	Number of terminar rays
5	Length of (the longest) terminal ray, cm
6	Number of branching of (the longest) terminal ray
	**Axillary rays**
7	Number of fertile axillary rays
8	Length of (the longest) fertile axillary ray, cm
	**Middle stem leaf**
9	Length of a middle stem leaf, cm
10	Width of a middle stem leaf, cm
11	Ratio Length of a middle stem leaf / Width of a middle stem leaf
12	Distance from the base to the widest part of a middle stem leaf, cm
13	Ratio Distance from the base to the widest part of a middle stem leaf / Length of a middle stem leaf
	**Ray leaves**
14	Length of a ray leaf, cm
15	Width of a ray leaf, cm
16	Ratio Length of a ray leaf/Width of a ray leaf
17	Distance from the base to the widest part of a ray leaf, cm
18	Ratio Distance from the base to the widest part of a ray leaf / Length of a ray leaf
	**Raylet leaves**
19	Length of a raylet leaf, cm
20	Width of a raylet leaf, cm
21	Ratio Length of a raylet leaf/Width of a raylet leaf
22	Distance from the base to the widest part of a raylet leaf, cm
23	Ratio Distance from the base to the widest part of a raylet leaf / Length of a raylet leaf
	**Cyathium**
24	Length of cyathial involucre, mm
25	Width of cyathial involucre, mm
26	Ratio Length of cyathial involucre/Width of cyathial involucre
27	Depth of gland emargination, mm
28	Length of cyathial gland, mm
29	Width of cyathial gland, mm
30	Ratio Depth of gland emargination/Length of cyathial gland
31	Ratio Length of cyathial gland/Width of cyathial gland
	**Fruit**
32	Fruit length, mm
33	Fruit width, mm
34	Ratio Fruit length/Fruit width
35	Distance from the base to the widest part of the fruit, mm
36	Ratio Distance from the base to the widest part of the fruit/Fruit length
37	Style length, mm
38	Fruit glabrous/pubescent/glandular
	**Seed**
39	Seed length, mm
40	Seed width, mm
41	Ratio Seed length/Seed width
42	Distance from the base to the widest part of a seed, mm
43	Ratio Distance from the base to the widest part of a seed/Seed length
44	Caruncle length, mm
45	Caruncle width, mm
46	Ratio Caruncle length/Caruncle width
47	Distance from the base to the widest part of caruncle, mm
48	Ratio Distance from the base to the widest part of caruncle/Caruncle length

## Results

### Phylogenetic and Biogeographic Analyses Based on the Complete RADseq Dataset

**FIGURE 3:**
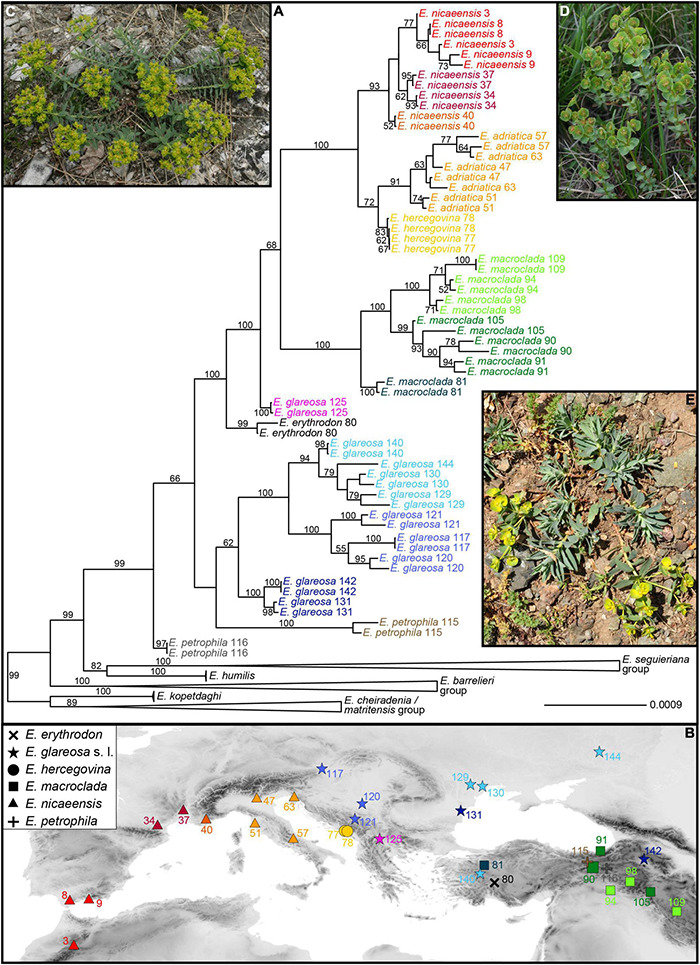
**(A)** Phylogenetic relationships within the *Euphorbia nicaeensis* alliance and between this alliance and its closest relatives within *E.* sect. *Pithyusa*, as inferred by maximum likelihood analysis of RADseq loci, with indicated bootstrap values above 50%. **(B)** Geographic provenance of the investigated populations with colour coding as in **(A)**. Two outgroup species, *E. seguieriana* (from Austria; **C**) and *E. triflora* (from Slovenia; **D**) from the *E. seguieriana* and the *E. barrelieri* groups, and the ingroup *E. petrophila* (from Turkey; **E**) are shown *in situ*. Photos: B. Frajman **(C,D)**, C. Gilly **(E)**. Population numbers correspond to Supplementary Table 1. The tree, including outgroup taxa, is presented in Supplementary Figure 2.

The average number of raw single reads per sample retained after quality filtering was ca. 0.74 million (standard deviation, *SD* = 0.3). The RADseq data are available in the NCBI Short Read Archive (SRA; BioProject ID PRJNA761526, BioSample accessions SRR15817339-SRR15817453). The relationships inferred with RAxML, based on 18,059 SNPs (5,731 variable loci), reflected both the taxonomic entities (Figure 3A and Supplementary Figure 2) and the geographic structure (Figure 3B). The *E. barrelieri-nicaeensis-seguieriana* clade was monophyletic (bootstrap support, BS, 99%). The *E. barrelieri* group, including *E. barrelieri*, *E. kerneri* Huter, *E. saxatilis* Jacq., *E. thessala* (Formánek) Degen and Dörfl. and *E. triflora* Schott, Nyman and Kotschy (Figure 3D; BS, 100%), was sister to a clade (BS 99%), consisting of our study group taxa (BS 99%) and a sister lineage (BS 82%), consisting of *E. humilis* (BS, 100%), as well as *E. niciciana* Borbás and *E. seguieriana* Neck. (*E. seguieriana* group; BS, 100%; Figure 3C).

Within our study group *E. petrophila* 116 (Figure 3E) was sister to all other accessions (BS, 66%), which formed three lineages: the first corresponded to *E. petrophila* 115, the second comprised most accessions of *E. glareosa*s.l. (BS, 62%), and the third included all other accessions in a clade with BS of 100%. In this latter clade, *E. erythrodon* 80 and *E. glareosa* 125 were consecutive sisters to a clade with all other accessions (BS, 68%), consisting of *E. macroclada* (BS, 100%) and *E. nicaeensis*, including *E. hercegovina* (BS, 100%). Within the main clades, corresponding to *E. glareosa* s.l., *E. macroclada* and *E. nicaeensis* (including *E. hercegovina*), relationships mostly reflected geography (Figure 3B), notable exceptions being geographically distant populations 131 and 142 of *E. glareosa*s.l. that were grouped together in a clade with the BS of 100%, and the population 105 of *E. macroclada*, which was geographically amongst the phylogenetically more divergent populations. Populations of *E. nicaeensis* were included in two clades, of which the western Mediterranean clade (BS, 93%) was sister to a clade (BS, 72%), including *E. hercegovina* (BS, 83%) and the Apennine and northwesternmost Balkan populations of *E. nicaeensis* (BS, 91%). To increase readability, from here on, as well as in the figures and tables, we refer to the western Mediterranean populations as *E. nicaeensis*, to the Apennine-Balkan populations as *E. adriatica*, and to both together as *E. nicaeensis* s.l.

The time-calibrated species tree of the entire dataset (Supplementary Figure 3 and Figure 2B) inferred similar main relationships as RAxML, but with certain differences. The *E. barrelieri* group (PP 1) included *E. barrelieri*, *E. kerneri*, *E. thessala*, and *E. triflora* (PP 1), but not *E. saxatilis*, which was resolved as sister (0.96 PP) to the *E. seguieriana* group (PP, 1). The *E. nicaeensis* alliance was weakly supported as monophyletic (PP, 0.90). Relationships among monophyletic (PP, 1) *E. adriatica*, *E. hercegovina*, and *E. nicaeensis* were unresolved, as was their relationship to *E. glareosa* and *E. macroclada*. They all formed a clade (PP, 1), a sister to *E. petrophila*. Relationships amongst the three main groups of the *E. barrelieri-nicaeensis-seguieriana* clade (including *E. humilis*) were poorly supported; the onset of their diversification dated back to 3.3 Ma (95% HPD, 1.8–4.8 Ma). The *E. nicaeensis* alliance putatively originated 2.8 Ma (95% HPD, 1.6–3.8 Ma); its diversification might have started in the early Pleistocene, 2.2 Ma (95% HPD, 1.1–3.2 Ma), and continued throughout the Pleistocene until *E. adriatica*, *E. hercegovina*, and *E. nicaeensis* might have diverged in the late Pleistocene, 0.4–0.6 Ma (95% HPD, 0.1–1.1 Ma).

As a result of comparison amongst six biogeographic models using BioGeoBears, the DEC + J model was selected as the best fitting. The best model not including the parameter +J (which was questioned by Ree and Sanmartin, 2018) was DEC. Detailed comparison of the models is given in Supplementary Table 3. Ancestral-area estimation with BioGeoBears (Supplementary Figure 3 and Figure 2B) indicated a high uncertainty in the geographic origin of the entire *E. barrelieri*-*nicaeensis*-*seguieriana* clade but indicated with high probability that the MRCA of the *E. nicaeensis* alliance was distributed either throughout Anatolia (area C; DEC + J) or Anatolia and the eastern Mediterranean (areas B and C; DEC). While *E. macroclada* and *E. petrophila* remained in Anatolia and adjacent regions of the IT, *E. glareosa* s.l. extended its distribution to the Pannonian and Pontic steppic areas, and the MRCA of the *E. nicaeensis* group dispersed further west into the Mediterranean Basin, where it diverged, giving rise to the western Mediterranean *E. nicaeensis* and the eastern Mediterranean *E. adriatica* and *E. hercegovina*.

### Phylogenetic Analyses Based on the Reduced RADseq Dataset for the *Euphorbia nicaeensis* Alliance

**FIGURE 4:**
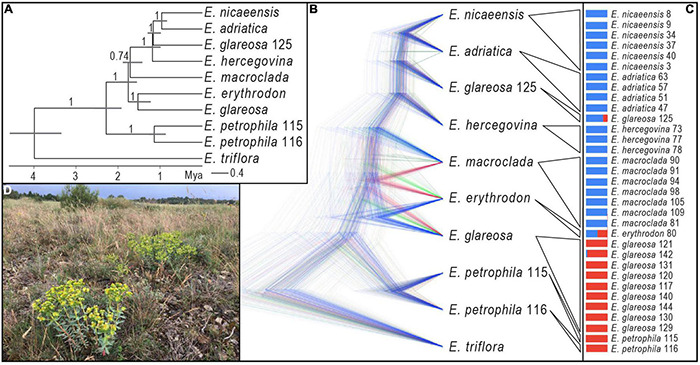
Phylogenetic relationships within the *Euphorbia nicaeensis* alliance based on RADseq data. **(A)** The time-calibrated species tree inferred with SNAPP. Numbers above branches are posterior probabilities, and the horizontal bars correspond to 95% highest posterior densities (HPD) of the age estimates. **(B)** Alternative topologies visualised with DensiTree and represented by different colours. **(C)** Division of all populations into two groups (blue and red) with Bayesian clustering using fastSTRUCTURE. **(D)** *Euphorbia nicaeensis* in its natural habitat northwest of Carcassonne in France (Photo: B. Frajman). Population numbering corresponds to Supplementary Table 1.

The species tree of the *E. nicaeensis* alliance with *E. triflora* as the outgroup based on 3,000 SNPs (Figure 4A) revealed a slightly different topology compared to the analyses of the complete dataset. The monophyly of the *E. nicaeensis* alliance was well supported (1 PP), and the onset of its diversification was estimated at 2.3 Ma (95% HPD, 1.9–2.5 Ma). Contrary to the analyses of the complete dataset with RAxML, *E. petrophila* was monophyletic (PP, 1) and was a sister to all other ingroup taxa. *Euphorbia erythrodon* was supported as a sister to *E. glareosa* (PP1; the *E. glareosa* group), and both were a sister (PP 1) to a poorly supported clade (PP, 0.74; the *E. nicaeensis* group), comprising all other taxa and population 125 of *E. glareosa*. The split between the *E. glareosa* group and the *E. nicaeensis* group was dated at 1.8 Ma (95% HPD, 1.6–2 Ma). Within the *E. nicaeensis* group, *E. macroclada* was a sister to all other species that formed a monophyletic group (PP, 1), with the onset of the diversification dated at 1.2 Ma (95% HPD, 1–1.3 Ma). In the latter group, *E. nicaeensis* (Figure 4D) was a sister to *E. adriatica* (PP, 1), both were a sister to *E. glareosa* 125 (PP, 1), and all three were sisters to *E. hercegovina* (PP, 1).

Bayesian clustering (Figure 4C) split the *E. nicaeensis* alliance into two clusters, one including *E. adriatica*, *E. hercegovina*, *E. macroclada*, and *E. nicaeensis*, and the other one, *E. petrophila*, and most populations of *E. glareosa*. Two populations were admixed between both clusters, namely, *E. erythrodon* 80 and *E. glareosa* 125. A conflicting position of *E. erythrodon*, between *E. glareosa* and *E. macroclada*, was also suggested by the occurrence of alternative topologies in the SNAPP trees, as visualised with DensiTree (Figure 4B); otherwise, the topology corresponded to the MCC tree (Figure 4A).

### Internal Transcribed Spacer Phylogenies

**FIGURE 5:**
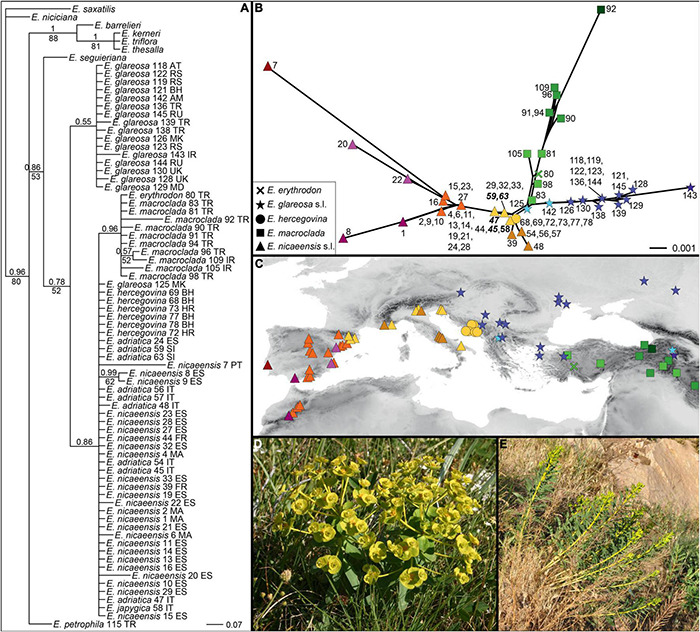
Phylogenetic relationships within the *Euphorbia nicaeensis* alliance and between this alliance and its closest relatives within *E.* sect. *Pithyusa* as inferred by Internal Transcribed Spacer (ITS) sequences. **(A)** Bayesian consensus phylogram with posterior probabilities > 0.50 above, and parsimony bootstrap values > 50% below branches; country codes follow the accession names. **(B)** NeighborNet and **(C)** geographic position of the ITS ribotype groups revealed by the NeighborNet and indicated by different colours. Population numbers in **(A,B)** are presented in Supplementary Figure 1 and in Supplementary Table 1. Population numbers of populations that are based on our revised taxonomic treatment belong to *E. adriatica* and are in **(B)** in bold italics (45, 47, 59, 63), and the one corresponding to *E. japygica* (58) is in bold. **(D)** *Euphorbia adriatica* from Italy and **(E)** *E. macroclada* from Turkey in their natural habitats. Photos: B. Frajman **(D)**, C. Gilly **(E)**.

Of 715 characters included, 33 (4.6%) were parsimony informative; consistency and retention indices were 0.87 and 0.96, respectively. The trees inferred by parsimony and Bayesian analyses (Figure 5A) were largely congruent, with the exception of relationships within the ingroup, which remained unresolved with parsimony. In general, the ingroup (excluding *E. petrophila*) was poorly supported as monophyletic (PP, 0.78; MPB, 52%) and included two unsupported clades in the Bayesian tree, the first corresponding to *E. glareosa*s 0.l (PP, 0.55) and the second to the *E. nicaeensis* clade (PP, 0.86). This second clade included a polytomy, including *E. adriatica, E. hercegovina*, *E. japygica*, *E. nicaeensis*, and an accession of *E. glareosa* 125 from North Macedonia, as well as a clade (PP, 0.96), with *E. macroclada* and the only accession of *E. erythrodon*. *Euphorbia petrophila* was positioned amongst the outgroup taxa.

The NeighborNet (Figure 5B) revealed a geographic structure (Figure 5C) in the variation of ITS sequences. Accessions of *E. adriatica* (Figure 5D), *E. hercegovina*, *E. japygica*, and *E. nicaeensis* from the central Mediterranean were positioned in the centre of the network (yellow, brown). Three main splits branched from the central ribotypes, corresponding to the western Mediterranean *E. nicaeensis* (orange, red, violet), *E. glareosa* s.l. (blue), and *E. macroclada* (Figure 5E), including *E. erythrodon* (green).

### Relative Genome Size

**FIGURE 6:**
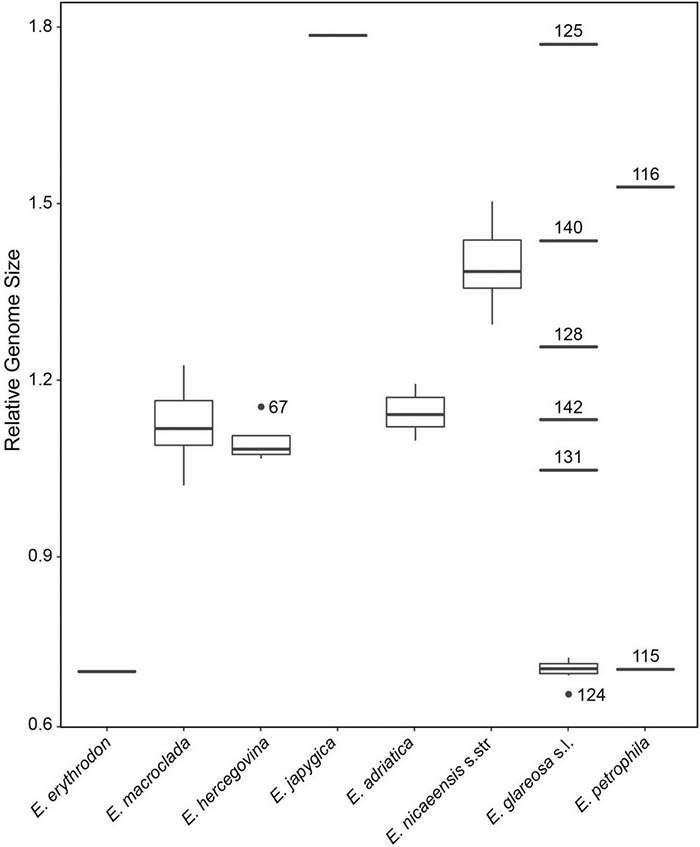
Relative genome size (RGS) variation in the *Euphorbia nicaeensis* alliance. Outliers putatively belonging to the same ploidy level as the majority of the samples are presented as dots, whereas putatively ploidy-divergent outliers are presented as lines, including their population numbers, which correspond to Supplementary Figure 1 and Supplementary Table 1.

Relative genome size values revealed clearly different DNA-ploidy levels (Suda and Trávníček, 2006) within *E. glareosa* s.l. and *E. petrophila*, whereas only diploids were found within *E. erythrodon*, *E. hercegovina*, *E. macroclada*, and *E. nicaeensis* s.l.; the single population sample of *E. japygica* was polyploid (Figure 6 and Supplementary Figure 4). The mean RGS for the single population sample of *E. erythrodon* was 0.71 and that of *E. macroclada* ranged between 1.02 and 1.23. In *E. hercegovina*, RGS ranged between 1.07 and 1.15. The RGS of *E. nicaeensis* s.l. ranged between 1.10 and 1.50 and was discretely distributed; the populations from the Apennine Peninsula and the northwesternmost Balkan Peninsula (*E. adriatica*) had RGS between 1.10 and 1.19, and those from west of the Alps (including the Maritime Alps in France) between 1.29 and 1.50. The only RGS-polyploid population from the *E. nicaeensis* lineage was that of *E. japygica* with RGS of 1.79. RGS-diploid populations of *E. glareosa* s.l. had RGS between 0.67 and 0.73, whereas the putatively polyploid populations had divergent RGS values, ranging between 1.05 and 1.77 (population, 131: RGS, 1.05; 142: 1.13; 128: 1.26; 140: 1.44; 125: 1.77). The two populations of *E. petrophila* had RGS of 0.71 (likely diploid) and 1.53 (likely tetraploid).

### Morphometry

The character-states for all morphological characters, including ratios, are presented in Supplementary Table 2. Box plot diagrams of important differential characters are shown in Supplementary Figure 5. The correlation coefficients exceeded 0.9 in two character pairs: Length of a middle stem leaf/Distance from the base to the widest part of a middle stem leaf and Fruit length/Fruit width, and, thus, the characters Distance from the base to the widest part of a middle stem leaf and Fruit width were excluded from the PCA and DA analyses.

**FIGURE 7:**
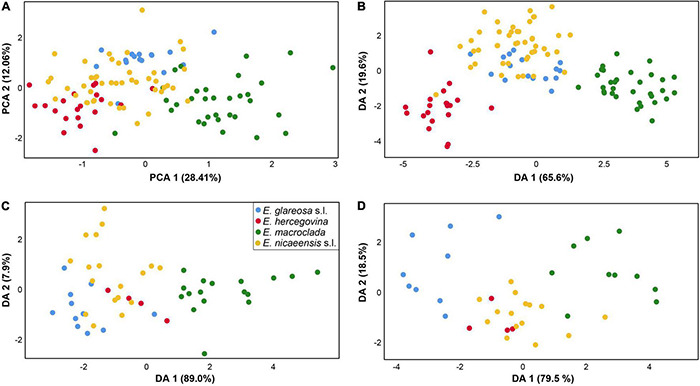
Morphological differentiation amongst *Euphorbia glareosa* s.l. (blue), *E. hercegovina* (red), *E. macroclada* (green), and *E. nicaeensis* s.l. (yellow). Principal component analysis (PCA; **A**) and discriminant analyses (DA) based on **(B)** 21 metric and nine ratio vegetative and cyathium characters, **(C)** four metric and two ratio fruit characters, and **(D)** six metric and four ratio seed characters.

The PCA scatter plot (first three components explaining 28.41, 12.06, and 8.65% of the total variation) based on vegetative and cyathium characters showed a weak separation of *E. macroclada* from *E. nicaeensis* s.l., *E. glareosa* s.l., and *E. hercegovina*, along the first component but a big overlap among all four species on the second component (Figure 7A). The characters, which contributed most to the separation along the first component, i.e., those having the highest component scores (between 0.65 and 0.86) were stem length, stem width, length of the longest terminal ray, length of the longest fertile axillary ray, length of a middle stem leaf, width of a middle stem leaf, length of a ray leaf, width of a ray leaf, length of a raylet leaf, and width of a raylet leaf. The DA scatter plot (Figure 7B) showed a clear separation of both *E. macroclada* and *E. hercegovina* from the other two taxa (*E. glareosa* s.l. and *E. nicaeensis* s.l.) along the first factor (Wilks’ Lambda = 0.019, χ^2^ = 378.856, df = 90, *p* < 0.001) and an overlap amongst all four species along the second factor (Wilks’ Lambda = 0.138, χ^2^ = 187.835, df = 58, *p* < 0.001). Variables with the highest discriminant loadings on the first factor were stem width, length of a ray leaf, length of a raylet leaf, width of a raylet leaf, width of cyathial involucre, ratio length of cyathial involucre/width of cyathial involucre, depth of gland emargination, length of cyathial gland, width of cyathial gland, and ratio length of cyathial gland/width of cyathial gland. BoxPlots (Supplementary Figure 5) revealed that *E. macroclada* is the largest of the studied species, resulting in higher measurement values of characters indicated as important in PCA and DA analyses. On the other hand, *E. hercegovina* has the smallest and narrowest leaves of all four taxa; the cyathial involucre is narrowest in *E. glareosa* s.l., which, consequently, has the highest ratio between length and width of the cyathial involucre.

For fruit characters, the PCA (first three components explaining 42.30, 29.18, and 16.64% of the total variation) showed a weak separation of *E. macroclada* from all other species along the first component but a strong overlap along the second component (not shown). The characters contributing the most to the separation along the first component, i.e., those having the highest component scores (between 0.8 and 0.99), were fruit length, distance from the base to the widest part of the fruit and style length. Similarly, the DA scatter plot (first factor: Wilks’ Lambda = 0.142, χ^2^ = 85.856, df = 18, *p* < 0.001; second factor: Wilks’ Lambda = 0.667, χ^2^ = 17.801, df = 10, *p* = 0.058) showed a separation of *E. macroclada* from *E. glareosa* s.l., *E. hercegovina*, and *E. nicaeensis* s.l. along the first factor but an overlap amongst all four species along the second factor, where a slight trend separating *E. glareosa* s.l. and *E. nicaeensis* s.l. could be observed (Figure 7C). The characters fruit length and distance from the base to the widest part of the fruit had the highest discriminant loadings on the first factor. BoxPlots (Supplementary Figure 5) confirmed that *E. macroclada* had the largest fruits, resulting in larger measurement values for most fruit characters. They also indicated that *E. glareosa* s.l. can have smaller, especially narrower fruits compared to *E. nicaeensis* s.l., whereas the values of *E. hercegovina* overlapped with those of *E. nicaeensis* s.l.

The PCA for seed characters (first three components explaining 46.81, 20.41, and 15.03% of the total variation) showed an overlap between *E. nicaeensis* s.l., *E. hercegovina*, and *E. glareosa* s.l., and a weak separation of *E. macroclada* along the first component, whereas, along the second component, all species overlapped strongly (not shown). The characters contributing the most to the separation along the first component, i.e., those having the highest component scores (between 0.65 and 0.97), were seed length, seed width, distance from the base to the widest part of a seed, caruncle length, and caruncle width. The DA scatter plot (first factor: Wilks’ Lambda = 0.089, χ^2^ = 72.600, df = 30, *p* < 0.001; second factor: Wilks’ Lambda = 0.460, χ^2^ = 23.310, df = 18, *p* = 0.179) showed an overlap between *E. nicaeensis* s.l. and *E. hercegovina* and a slight separation of *E. glareosa* s.l. and *E. macroclada* along the first factor, and an overlap amongst all taxa along the second factor (Figure 7D). Variables with highest discriminant loadings on the first factor were seed length, ratio seed length/seed width, ratio of distance from the base to the widest part of a seed/seed length, caruncle length, caruncle width, and ratio caruncle length/caruncle width.

**FIGURE 8:**
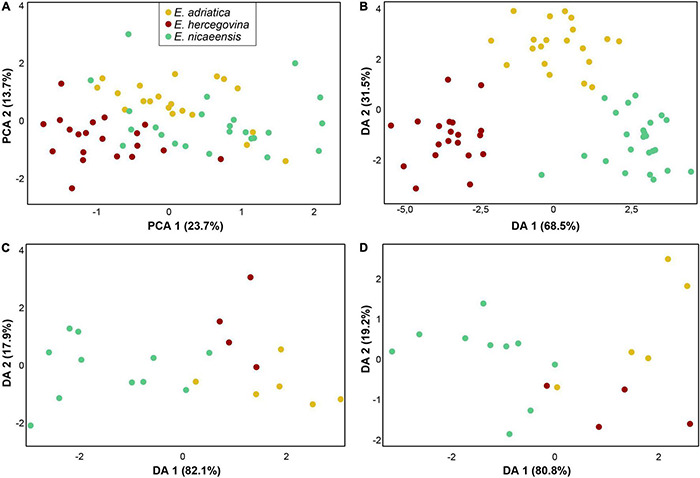
Morphological differentiation amongst *Euphorbia adriatica* (yellow), *E. hercegovina* (red), and *E. nicaeensis* (green). Principal component analysis (PCA; **A**) and discriminant analyses (DA) based on **(B)** 21 metric and nine ratio vegetative and cyathium characters, **(C)** five metric and two ratio fruit characters, and **(D)** six metric and four ratio seed characters.

The PCA and DA analyses, including only closely related *E. adriatica*, *E. hercegovina*, and *E. nicaeensis*, showed a separation amongst the three taxa that were most pronounced in the vegetative and cyathium characters (Figure 8A,B) and less pronounced in fruit and seed characters (Figure 8C,D). The PCA scatter plot (first three components explaining 23.72, 13.74, and 10.76% of the total variation) based on vegetative and cyathium characters showed a slight trend in separation of the three taxa but also a strong overlap (Figure 8A). On the other hand, the DA scatter plot (Figure 8B) showed a clear separation amongst the three taxa. Variables with the highest discriminant loadings on the first factor (Wilks’ Lambda = 0.030, χ^2^ = 162.458, df = 60, *p* < 0.001), which clearly separated *E. hercegovina* from *E. nicaeensis*, whereas *E. adriatica* was intermediate, were length of (the longest) terminal ray, number of fertile axillary rays, length of (the longest) fertile axillary ray, length of a middle stem leaf, width of a middle stem leaf, ratio length of a middle stem leaf/width of a middle stem leaf, width of a ray leaf, ratio length/width of a ray leaf, distance from the base to the widest part of a ray leaf, ratio of distance from the base to the widest part of a ray leaf/length of a ray leaf, length of a raylet leaf, width of a raylet leaf, ratio length/width of a raylet leaf, distance from the base to the widest part of a raylet leaf, ratio of distance from the base to the widest part of a raylet leaf/length of a raylet leaf, width of cyathial involucre, and ratio length of cyathial involucre/width of cyathial involucre. Variables with highest discriminant loadings on the second factor (Wilks’ Lambda = 0.240, χ^2^ = 66.352, df = 29, *p* < 0.001), which separated *E. adriatica* from the other two species, were stem glabrous/pubescent, length of (the longest) terminal ray, width of a middle stem leaf, ratio length of a middle stem leaf/width of a middle stem leaf, length of a ray leaf, width of a ray leaf, distance from the base to the widest part of a ray leaf, length of a raylet leaf, width of a raylet leaf, ratio length/width of a raylet leaf, length of cyathial involucre, width of cyathial involucre, ratio length of cyathial involucre/width of cyathial involucre, depth of gland emargination, length of cyathial gland, ratio depth of gland emargination/length of cyathial gland, and ratio length of cyathial gland/width of cyathial gland.

The PCAs of fruit and seed characters, respectively, showed a high overlap of *E. adriatica*, *E. hercegovina*, and *E. nicaeensis* (not shown), whereas the DAs (Figure 8B,C) indicated a discrimination trend between *E. nicaeensis* from *E. adriatica* and *E. hercegovina* along the first factors (fruit characters: Wilks’ Lambda = 0.175, χ^2^ = 26.134, df = 14, *p* = 0.025; seed characters: Wilks’ Lambda = 0.204, χ^2^ = 19.089, df = 18, *p* = 0.386). Variables with highest discriminant loadings on these factors were fruit length, fruit width, ratio fruit length/fruit width, seed length, seed width, ratio seed length/seed width, distance from the base to the widest part of a seed, ratio of distance from the base to the widest part of a seed/seed length, caruncle length, and distance from the base to the widest part of caruncle.

## Discussion

RADseq, originally developed for intraspecific phylogeographic studies (McCormack et al., 2013; Lemmon and Lemmon, 2013), allowed us to establish a clear phylogenetic hypothesis regarding the origin of and the relationships within the *E. nicaeensis* alliance. The RADseq data clearly inferred the *E. nicaeensis* alliance as monophyletic (Figures 2, 3) and, alongside the RGS and morphometric data, helped us describe a new species, *E. adriatica* (see below), which is, together with *E. erythrodon*, *E. glareosa* s.l., *E. hercegovina*, *E. japygica, E. macroclada*, *E. nicaeensis*, and *E. petrophila*, a member of this alliance.

### Phylogeographic Origin and Diversification of the Mediterranean Taxa

Within the Mediterranean Basin, diversification patterns revealed by different methods indicate complex evolutionary pathways and cryptic divergence that went unnoticed by earlier taxonomists. Biogeographic analyses (Figure 2B) support the Western Asian origin of the Mediterranean lineage, which diversified *in situ* likely as a result of Pleistocene climatic oscillations, accompanied by adaptation to different habitats and polyploidisation. Thus, our study underlines the importance of the IT floristic region as a source area for many Mediterranean lineages (Manafzadeh et al., 2017). Unexpectedly, one of the main genetic breaks revealed by RADseq (Figures 3, 4), accompanied also by a clear divergence in RGS (Figure 5), falls within the seemingly continuous distribution of *E. nicaeensis* s.l. and separates the populations west of the Alps from those south of the Alps, which we describe as a new species, *E. adriatica* (see below). Given the Pleistocene diversification within the *E. nicaeensis* alliance (Figures 2B, 4A), It is likely that the inferred phylogeographic pattern—also reflected in RGS and morphological divergence (Figures 6, 8)—is a result of survival in isolated Pleistocene glacial refugia in the western (Iberian Peninsula) and the central/eastern Mediterranean (Apennine and Balkan peninsulas). All three peninsulas are renowned as important glacial refugia, where distinct genetic lineages persisted through the Pleistocene and Quaternary climatic fluctuations (Bilton et al., 1998; Hewitt, 1999, 2000, 2011; Petit et al., 2003; Nieto Feliner, 2014; Cresti et al., 2019).

Interestingly, the western lineage (*E. nicaeensis*) largely corresponds in its range to *E. flavicoma* DC. from the *E. verrucosa* alliance, which was also suggested to have had its Pleistocene refugium in the Iberian Peninsula (Caković et al., 2021). *Euphorbia flavicoma* and *E. nicaeensis* are ecologically similar, inhabiting Mediterranean scrublands, dry and warm grasslands, and open forests. From the putative Pleistocene refugium in the Iberian Peninsula, both species extended their ranges to southern France, where their eastward migration was likely obstructed by the Alps. Similar moderate expansion out of Iberia has also been observed in *Arabis scabra* L. (Koch et al., 2020), *Pinus pinaster* Aiton (Bucci et al., 2007), and *Quercus suber* L. (Magri et al., 2007). Congruent expansion patterns of different warm-adapted taxa have likely been influenced by climatic factors, which prevented more extensive dispersals out of Iberia (Caković et al., 2021). In westernmost Europe, Mediterranean climate is, nowadays, prevalent in the Iberia, and is restricted to southernmost France (Peel et al., 2007).

Compared to *E. nicaeensis*, Apennine-Balkan *E. adriatica* exhibits a smaller RGS and is ecologically divergent, usually thriving in mesophilic submediterranean grasslands and scrublands in the Apennine Peninsula, the southern outskirts of the Alps, and the northwestern Balkan Peninsula. This species likely had its glacial refugium in the Apennine Peninsula, from where it dispersed to the southern margin of the Alps, which would have acted as a prominent biogeographic barrier for northward migration of Apennine biota (Hewitt, 2000). The third Mediterranean species, separated by a distribution gap of 300 km from *E. adriatica*, is the Balkan endemic *E. hercegovina*. It grows in open dolomitic grasslands and pine forests with a submediterranean character in the central Balkan Peninsula, where it likely had its Pleistocene refugium, and from where it did not spread considerably. It is morphologically clearly divergent and also has a divergent RGS. Contrary to many other examples of disjunctly distributed amphi-Adriatic lineages, in which the distribution area in the Balkan Peninsula exceeds in its size that in the Apennine Peninsula [see Frajman and Schönswetter (2017) and Falch et al. (2019) for reviews], this is not the case for *E. adriatica* and *E. hercegovina*. Partly incongruent relationships amongst the three species, resolved by different analytical approaches of the RADseq data outlined in the Results, are accompanied by different patterns of morphological and RGS divergence. Whereas the RGS data support a closer relationship between *E. adriatica* and *E. hercegovina* (Figure 6), morphological data point to *E. adriatica* and *E. nicaeensis* as more similar (Figure 8).

In addition to the Pleistocene glaciations, which fragmented the range of the Mediterranean *E. nicaeensis* lineage and likely triggered its divergence in three glacial refugia, adaptation to different substrates and climatic conditions, as well as polyploidisation in the southern Apennine Peninsula, contributed to diversification of the *E. nicaeensis* group. Specifically, the only analysed population of *E. japygica* from the southern Apennine Peninsula is DNA-polyploid (Figure 6). This, alongside the morphological differentiation reported by Fenu et al. (2016) and the lack of overlap in distribution with *E. adriatica* warrant recognition of this taxon at the species level, as originally proposed by Tenore (1830). Further studies, including more populations, are, however, needed to clarify the status of this taxon.

### Morphological Diversification Reflects Ecology Rather Than Phylogenetic Relationships

Morphological diversification only partly follows evolutionary trajectories in the *E. nicaeensis* alliance. The discordant patterns likely result from adaptation to similar habitats within divergent phylogenetic lineages, on the one hand, and to different habitats within the same evolutionary lineages, on the other hand. The traditional, morphology-based taxonomic treatments largely do not reflect evolutionary relationships. *Euphorbia glareosa* s.l. and *E. nicaeensis* s.l., which are morphologically similar (Figure 7) and were often considered conspecific (e.g., Radcliffe-Smith and Tutin, 1968; Kuzmanov, 1979; Radcliffe-Smith, 1982; Greuter et al., 1986; Govaerts et al., 2000), are, in fact, phylogenetically clearly divergent. Either adaptation to similar environments has triggered a parallel evolution of similar morphological traits in both lineages or the overall similarity of *E. glareosa* s.l. and *E. nicaeensis* s.l. was inherited from a shared common ancestor. It is well-known that the European steppes share several characteristics with the Mediterranean grasslands, which is reflected in multiple shared taxa and similar adaptations across both biogeographic regions (Peart, 2008; Hamasha et al., 2012). There is much variability in morphological traits connected to a habit (plant and leaf size) both within *E. nicaeensis*, but, especially, within *E. glareosa* s.l., which is reflected in the description of several intraspecific taxa (e.g., Kuzmanov, 1979; Greuter et al., 1986; Geltman, 2009, 2020). Whether, in the latter case, the morphological variability reflects evolutionary entities or, rather, adaptation to divergent habitats requires further investigation and is beyond the scope of this study.

Similarly, morphologically distinct *E. hercegovina*, which was earlier considered to belong to *E. barrelieri* (Hayek, 1924; Kuzmanov, 1963; Poldini, 1969; Greuter et al., 1986; Govaerts et al., 2000; Geltman, 2009), is nested within (Figure 3A) or closely related to *E. nicaeensis* s.l. (Figure 4). Its morphological divergence is likely a result of adaptation to dolomitic substrates, which are also typical for taxa of the *E. barrelieri* group (Frajman and Schönswetter, 2017). The soil derived from dolomitic bedrock is shallow and dry, resulting in extreme growing conditions. Plants growing in such habitats have to be tolerant of high magnesium and low-moisture levels, leading to the strong selective pressures that such extreme habitats impose (Ware, 1990; Noss, 2012).

Similarly, *E. erythrodon* growing on mountain ridges and screes has a dwarf prostrate habit, with stems that rarely exceed 7 cm (Radcliffe-Smith, 1982), which is likely an adaptation to mountain habitats (Larcher et al., 2010; Körner, 2012; Gehrke et al., 2016). A superficially similar case is provided by *E. seguieriana* subsp. *loiseleurii* (Rouy) Greuter and Burdet, which occurs in the windswept summit area of Mt. Ventoux in the French Provence, and exhibits a similar dwarf habit as an adaptation to this habitat. Whereas this latter taxon is nested within *E. seguieriana* and does not deserve taxonomic recognition (Frajman et al., 2019), *E. erythrodon* is phylogenetically distinct. However, the single studied population is resolved as intermediate between *E. glareosa* s.l. and *E. macroclada* (Figures 4B,C); further studies, including additional populations, are needed to confirm our preliminary findings. Finally, the more robust habit and bigger size of all organs in *E. macroclada*, as compared to *E. nicaeensis* s.l. (Supplementary Figure 5), are, possibly, a consequence of the former taxon mostly growing in deeper clay soils over siliceous and sandstone substrates, which are more humid and nutrient-rich than the better-drained calcareous substrates, on which *E. adriatica* and *E. nicaeensis* usually occur (Frajman, personal observation).

Altogether, our results demonstrate how heterogeneous environments can outweigh a phylogenetic signal, resulting in taxonomic treatments not reflecting evolutionary pathways. Also, in other plant lineages, It has been shown that heterogeneous environments have contributed to the high diversity of the Mediterranean (e.g., Frajman and Oxelman, 2007; Du Pasquier et al., 2017; Ðurović et al., 2017).

### Higher Phylogenetic and Relative Genome Size Diversity in Irano-Turanian and Steppic Areas as Compared to the Mediterranean Basin

The Mediterranean Basin acted as a cradle for the diversification of the *E. nicaeensis* lineage, where the phylogenetic relationships clearly reflect geographical distribution of the taxa. The diversification of the closely related taxa from the easterly adjacent IT and Eurasian steppic regions, in contrast, was more turbulent and geographically less coherent, resulting in multiple, clearly divergent sympatric lineages as indicated by RADseq data (Figures 2, 3), as well as in the polyploidisation events indicated by the RGS data (Figure 5). Whereas we only detected a single polyploid population (58) from southern Italy within *E. nicaeensis* s.l. (two further polyploid populations with 2*n* = 56 have been reported from Spain; Vilatersana and Bernal, 1992), we recorded multiple RGS-divergent populations within *E. glareosa* s.l. and *E. petrophila*. They possibly result from several polyploidisation events, even if other factors influencing changes in RGS (Pellicer et al., 2018) cannot be ruled out in the absence of chromosome counts.

The species from the IT region that appears most closely related to the Mediterranean taxa is *E. macroclada*, distributed from Anatolia to the Armenian, Iranian, and Syrian highlands (Figure 1; Radcliffe-Smith, 1982). Less clear is the phylogenetic position of narrowly distributed *E. erythrodon*, which is limited to mountain ridges and screes of southwestern and central Anatolia. It was resolved as a sister to the *E. nicaeensis* lineage by the complete RADseq dataset (Figure 3) and is nested within *E. macroclada* in the ITS phylogeny (Figure 5). The RADseq data limited to the *E. nicaeensis* alliance indicate its close relation to *E. glareosa* s.l. (Figure 4A), but also evidence admixture with sympatric *E. macroclada* (Figure 4B). A close relationship with *E. glareosa* s.l. is further supported by their similar RGS (Figure 6).

The RGS data (Figure 6) further indicate that a substantial increase in genome size (GS) likely happened in the common ancestor of *E. adriatica*, *E. hercegovina*, *E. macroclada*, and *E. nicaeensis*. Without chromosome counts, it is impossible to say whether this increase was due to polyploidisation. Since the two published chromosome counts of *E. macroclada* and most counts of *E. nicaeensis* are 2*n* = 18, which are the same as those of diploid *E. glareosa* s.l. and outgroup *E. niciciana* and *E. seguieriana* (Rice et al., 2015), we hypothesise that the increase in GS in the *E. nicaeensis* lineage was not due to polyploidisation but likely due to other processes. Alongside polyploidy, accumulation of retrotransposons and other repetitive elements is considered main factors of GS increase in angiosperms (Pellicer et al., 2018), e.g., leading to 2-fold increase in GS in the wild rice relative *Oryza australiensis* (Piegu et al., 2006). Genç and Kültür (2020), recently, have also published a tetraploid chromosome count from *E. macroclada*, whereas our RGS data only indicated polyploidisation in *E. glareosa* and *E. petrophila* from Turkey. Additional studies with more complete taxonomic and denser geographic sampling are needed to display how important role polyploidisation played for the diversification of this group in the IT region.

Most of the studied populations of *E. glareosa* s.l., with the exception of population 125 from North Macedonia, formed a monophyletic group in the RADseq data, closely related to *E. erythrodon* and *E. macroclada* (Figures 2, 3). In the ITS tree, these populations formed a poorly supported clade (Figure 5A), while, in the ITS NeighborNet, they were positioned along the same split, where population 125 was at the basis of this split, close to the populations of *E. hercegovina* and *E. adriatica* (Figure 5B). Population 125 from North Macedonia had the highest RGS of all studied populations, indicating its polyploid origin, which is likely responsible for its divergent phylogenetic placement separated from the other populations of *E. glareosa*. As all geographically close Balkan populations clearly belong to *E. glareosa* s.l.—the closest diploid population, 126, being only 25 km away—we also included population 125 in this species. Alternatively, based on the strikingly similar RGS of the population 58 of *E. japygica* from southern Italy (Figure 6), we cannot exclude their common origin, as population 125 also has hairy fruits, which is the most important character distinguishing *E. japygica* from *E. nicaeensis* s.l. (Tenore, 1830). Biogeographic connections between southern Italy and the Balkan Peninsula have been evidenced in several other plant groups [see Frajman and Schönswetter (2017) for a review]. Further studies, including chromosome counts and extensive sampling in southern Italy, as well as in geographically intermediate Albania, where populations belonging to the *E. nicaeensis* alliance are only known from three localities in the north of the country (Barina, 2017), are needed.

In the same line, the polyploid origin of population 116 of *E. petrophila* is likely the reason for its divergence from the diploid population 115, as inferred by the complete RADseq data (Figure 3A). On the other hand, in the analyses of the *E. nicaeensis* alliance dataset, both populations were sisters to all other ingroup accessions (Figure 4). Within *E. glareosa* s.l., additional divergent RGS values of some populations scattered throughout the entire distribution area have been observed (Figure 5). It remains unclear whether this is due to tetraploidisation, followed by genome downsizing that differentially acted in geographically distant populations, or if accumulation of repetitive elements (Pellicer et al., 2018) is responsible for the observed pattern. Further studies, with extended geographic and taxonomic sampling, are needed to disentangle the diversification patterns within *E. glareosa* s.l., which is an assemblage of around ten described taxa (Prokhanov, 1949; Kuzmanov, 1979; Radcliffe-Smith, 1982), and to establish its relationships with *E. petrophila* and the Anatolian narrow endemics *E. pestalozzae* Boiss. and *E. pisidica* Hub.-Mor. and M. S. Khan (Radcliffe-Smith, 1982).

### Partly Incongruent Patterns Inferred by Different Analyses of the Restriction Site Associated DNA Sequencing Data

Different analytical approaches of the RADseq data resulted in partly incongruent patterns outlined in the sections “Results” and “Discussion” above, which is often the case in phylogenetic analyses of such data (Wagner et al., 2020; Cai et al., 2021; Rose et al., 2021; Hühn et al., 2022). Both biological as well as methodological factors can be responsible for the observed incongruences. Having in mind the group’s diversification onset in the Pleistocene, and the vast areas (from northwest Africa and Iberia to Central Asia) that it inhabits, rapid range expansions resulting in incomplete lineage sorting and secondary contacts amongst recently diverged lineages can be responsible for some of the observed incongruences (Cai et al., 2021; Rose et al., 2021). Genetic admixture evidenced for *E. erythrodon* and the population 125 of *E. glareosa* (Figure 4C) was another possible cause underlying incongruences. The population 125 had, along with *E. japygica*, the highest genome size of all samples (Figure 6) and may be of allopolyploid origin. The polyploid nature of some samples can strongly influence phylogenetic inference. Incongruence between two differently analysed datasets was thus observed in *Salix*, where one of the analysed species was of allopolyploid origin (Wagner et al., 2020). However, for merely reconstructing relationships, which is the focus of our study, the approach used in the present study has been shown to be valuable even in plant groups with high incidence of polyploidy (Záveská et al., 2019).

In addition, topological differences between the two different RADseq datasets (Figure 4 vs. Figure 5) may be attributable also to the various amounts of loci shared across the sampled taxa. In the complete dataset (Figure 4), the limited amount of loci shared (1,498 SNPs) across the broad range of species sampled had too little information to resolve relationships at deeper nodes. On the other hand, the 3,000 loci shared across species limited only to the *E. nicaeensis* alliance better resolved relationships amongst them, and the inferred phylogenies better reflected the morphological (e.g., grouping of *E. adriatica* and *E. nicaeensis*, as well as both populations of *E. petrophila* with strong support) and the RGS data (e.g., grouping of *E. erythrodon* with *E. glareosa*). This underlines that RADseq data are better suited to infer relationships amongst most closely related taxa, compared to deeper evolutionary nodes.

### Taxonomic Considerations and a Revised Taxonomic Treatment

**FIGURE 9:**
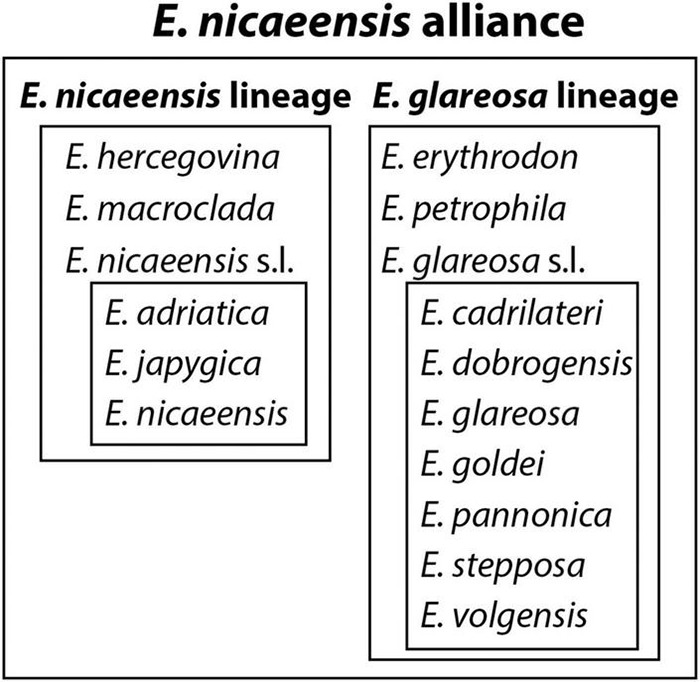
Relations amongst taxa within the *E. nicaeensis* alliance as a result of the outcomes of this study, combined with previous treatments within *E. glareosa* s.l., which were not addressed in this study.

The combination of phylogenetic, RGS, and morphometric data allows us to propose a revised taxonomic treatment of the *E. nicaeensis* alliance, resolving some long-standing uncertainties about species delimitations within this alliance, but also introducing new questions that will need to be answered in the future. In Figure 9, we graphically present the relations within the *E. nicaeensis* alliance, partly based on our study and partly outlined in the introduction (for *E. glareosa* s.l.). The main taxonomic outcomes of our study can be summarised in the following four points. (1) *Euphorbia nicaeensis*. l. and *E. glareosa* s.l. are phylogenetically divergent and geographically allopatric lineages with distinct RGS and should, despite their morphological similarity, be treated as distinct species and not as subspecies, as proposed by Radcliffe-Smith and Tutin (1968) and Greuter et al. (1986). (2) *Euphorbia macroclada* is a distinct species distributed in the IT region, closely related to the Mediterranean *E. nicaeensis* lineage, but including traces of genomic imprint shared with *E. erythrodon* and *E. glareosa*. (3) The Mediterranean *E. nicaeensis* lineage is an assemblage of three allopatric, phylogenetically divergent, and morphologically different (although with overlapping character states) groups of populations with distinct RGS that deserve recognition at the species level, namely, the western Mediterranean *E. nicaeensis*, the central-eastern Mediterranean *E. adriatica*, and the eastern Mediterranean *E. hercegovina*. In addition, we preliminary treat the southern Italian populations as a distinct species, *E. japygica*, but further studies are needed to explore whether all populations belonging to this taxon are (a) polyploid, (b) morphologically and phylogenetically distinct from *E. adriatica*, and (c) share a most recent common ancestor with Balkan populations in Albania and North Macedonia. (4) *Euphorbia erythrodon*, *E. glareosa* s.l., and *E. petrophila*, which thrive in the Eurasian steppes and the southerly adjacent IT region, need to be further studied based on an extensive geographic and taxonomic sampling (including also *E. pestalozzae* and *E. pisidica*) to (a) disentangle phylogenetic relationships and morphological differentiation amongst the populations and different taxa described from this area, (b) explore the incidence of polyploidy within this group, and (c) provide a revised taxonomic treatment.

#### Identification Key

High variability and overlap of morphological characters amongst the taxa of the *E. nicaeensis* alliance hinder a construction of a reliable identification key based solely on morphology. Especially, the overlap between *E. nicaeensis* s.l. and *E. glareosa* s.l. is considerable, whereas *E. hercegovina* and *E. macroclada* are more divergent. Moreover, absence or presence of horns on the cyathial glands, traditionally used to discriminate between *E. glareosa* s.l. and *E. macroclada*, respectively (e.g., Prokhanov, 1949; Radcliffe-Smith, 1982; Geltman, 2020), proved not to be a reliable character, as several examined specimens of *E. glareosa* s.l. also had horn-like appendages. Also the characters given in Flora Europaea (Radcliffe-Smith and Tutin, 1968) to distinguish between *E. glareosa* s.l. and *E. nicaeensis* s.l., i.e., the number of rays and capsule size, have proved to be highly overlapping between the two taxa (see species descriptions below). In the following key, several morphological characters, therefore, overlap, but a combination of several characters with geographic data makes unambiguous identification possible.

1   Robust erect plants, (19) 30–62 (70) cm high, with (3) 4–5 (6)-mm thick stems. Cauline leaves (3) 4–6.5 (8) × (0.5) 0.7–1.6 (2) cm. Cyathial glands with two, often lobate or multifid horns. Fruits (5.9) 6.1–8.4 (8.8) × (3.1) 4.1–5.7 (6.2) mm. Seeds (2.9) 3.1–4 (4.2) × (1.9) 2–2.5 (2.6) mm. *West Asia*………………………………………………….***E. macroclada.***
1* Less robust, decumbent to erect plants, (4) 10–40 (45)-cm high, with (1) 2–4-mm thick stems. Cauline leaves (0.7) 1.5–5 (5.5) × (0.2) 0.4–1.1 (1.5) cm. Cyathial glands without or with horns; horns sometimes lobate, never multifid. Fruits (3.6) 4–5.5 (6) × (2.2) 2.4–3.8 (4.2) mm. Seeds (2) 2.5–3.1 (3.4) × (1.2) 1.5–2 (2.1) mm……………… **2.**
2   Decumbent plants, (6.5) 8–17 (22)-cm high, with 1–2-mm thick stems. Cauline leaves (0.7) 1–2 (2.7)-cm long, (1.1) 1.6–4.1 (5.4) times longer than wide. *Central Balkan Peninsula*………………………………………………… ***E. hercegovina.***
2*  Decumbent to erect plants, (5) 11–40 (45)-cm high, with (1) 2–4-mm thick stems. Cauline leaves (1.7) 2–5 (5.5)-cm long, (2.3) 3.2–6.2 (6.8) times longer than wide…………….. **3.**
3   Terminal rays 5–9 (11), the longest (2) 2.5–5.5 (7)-cm long. Cauline leaves 1.8–4 (4.5) × (0.3) 0.4–0.8 cm, (3.8) 4.6–6.2 (6.8) times longer than wide. Fruits (3.6) 3.7–4.4 (4.6) mm × (2.3) 2.4–3.1 mm. Seeds, 2.6–2.7 mm × 1.6–1.9 (2) mm, (1.3) 1.4–1.6 (1.7) times longer than wide. *Apennine Peninsula*, *northern Balkan Peninsula*………………………….. **4.**
3* Terminal rays 5–12 (13), the longest (1.2) 3–8.5 (10)-cm long. Cauline leaves (1.7) 2.3–5 (5.5) cm × (0.5) 0.6–1.2 (1.5) cm, (2.3) 3.2–5.5 (6.5) times longer than wide. Fruits (3.6) 4.1–5.7 (6) mm × (2.2) 3–4.1 (4.2) mm. Seeds (2) 2.2–3.1 (3.4) mm × 1.2–2.1 mm, (1.4) 1.5–1.9 (2) times longer than wide. *Western Mediterranean (excl. Apennine Peninsula), Central and East Europe, West Asia*……………. **5.**
4   Fruits glabrous. *Northern and* central *Apennine Peninsula*, *northern Balkan Peninsula*…………………………… ***E. adriatica***
4* Fruits pubescent. *Southern Apennine Peninsula* ……………………………………………………………………… ***E. japygica.***
5   Fertile axillary rays (1) 2–9 (10), the longest (1.2) 2–7.2 (8.1)-cm long. Cauline leaves (1.7) 2.2–3.8 (5) cm × (0.5) 0.6–1 cm. Cyathial glands usually bigger, (0.7) 0.8–1.5 (1.7) mm × (0.9) 1.1–1.9 (2.1) mm. Fruits (4) 4.3–5.7 (6) mm × (2.7) 3.1–4.1 (4.2) mm, 1.3–1.5 (1.6) times longer than wide. Styles (0.9) 1.9–2.2-mm long. Seeds, 2.7–3.1 (3.3) mm × 1.8–2.1 mm. *Western Mediterranean*…………………………………………….. ***E. nicaeensis***
5* Fertile axillary rays (2) 4–14 (15), the longest (3.2) 3.9–8.9 (12.2)-cm long. Cauline leaves (2.4) 2.5–5 (5.3) × (0.5) 0.6–1.2 (1.5) cm. Cyathial glands usually smaller, 0.7–1 mm × 1–1.5 mm. Fruits (3.6) 4.1–4.6 (5.5) mm × (2.2) 2.4–3.1 (3.3) mm, 1.4–1.9 times longer than wide. Styles (1.1) 1.3–1.7-mm long. Seeds, (2) 2.2–3.1 (3.4) mm × (1.2) 1.3–1.7 (1.8) mm. *Central and East Europe, West Asia.* ……………………………………………………………………….***E. glareosa.***

#### Taxonomic Treatment

(1) ***Euphorbia adriatica* Stojilkovič, Záveská, and Frajman, sp. nov.** Type: Holotype: “Flora of Slovenia, Primorska, Kras: south of the road Senožeče – Senadole, 1.5 km west of Senožeče; 550 m; 14°0‘38′′ E 45°43′9′′ N; dry meadow. 16 August 2021 *V. Stojilkovič and B.* Frajman 16939 (W0164201^5^). Isotypes in IB 113154, LJU, FI018954, ZA 62967 and 62969).
= *E. seguieriana* var. *prostrata* Fiori, Nuov. Fl. Italia 2: 183. 1926 *≡E. nicaeensis* subsp. *prostrata* (Fiori) Arrigoni, Inform. Bot. Ital. 12: 140. 1980 (publ. 1981). — Type: Flora Italica – Herbarium Adr. Fiori: “Prov. di Firenze, Impruneta ai Sassi neri, solo serpentinoso, 315 m,” 4 June 1911, *Adr[iano] Fiori s.n.* (FI002664!).
*Note*: *Euphorbia nicaeensis* subsp. *prostrata* has been described from serpentine outcrops in Italy, and dwarf individuals from these populations are, in our opinion, merely ecotypes adapted to this specific substrate. A similar adaptation to serpentines, partly at the same localities, has been observed also in *Euphorbia spinosa* L. (Stevanoski et al., 2020).
*Diagnosis: Euphorbia adriatica* differs from closely related *E. hercegovina* in being more robust in all vegetative characters, i.e., mostly having higher and thicker stems and bigger leaves, but smaller fruits. Compared to *E. nicaeensis*, *E. adriatica* often has relatively longer leaves compared to their width and smaller fruits with shorter styles as well as smaller seeds. It also has glabrous fruits, whereas *E. japygica* has pubescent fruits.
*Description:* Glabrous and glaucous perennial, (7) 11–37 (40)-cm high, with (1) 2–3-mm thick stems. Terminal rays 5–9 (11), the longest (2) 2.5–5.5 (6.7)-cm long, 1–2 times dichotomously branched. Fertile axillary rays 2–7 (10), the longest (2) 2.6–6.1 (7.2)-cm long. All leaves with entire margin. Cauline leaves linear oblanceolate (1.8) 1.9–4.1 (4.4) cm × (0.3) 0.4–0.8 cm, (3.8) 4.6–6.2 (6.8) times longer than wide, widest at (0.6) 0.7–0.8 of their length, with cuneate base, and acute apex. Ray leaves broadly ovate, (0.7) 0.9–1.8 cm × 0.5–1.1 (1.2) cm, (1) 1.1–2.1 (2.7) times longer than wide, widest at 0.4–0.6 (0.7) of their length. Raylet leaves broadly ovate to reniform, 0.6–1 (1.3) cm × 0.8–1.4 (1.5) cm, (0.6) 0.7–0.8 (0.9) times longer than wide, widest at (0.1) 0.2–0.5 (0.5) of their length, with cordate base, and obtuse apex. Cyathial involucre campanulate, (1.6) 1.8–2.6 (2.7) mm × (1.4) 1.7–2.7 (3.2) mm, (0.8) 0.9–1.4 (1.6) times longer than wide. Cyathial lobes usually pubescent. Cyathial glands obovate-truncate, (0.6) 0.7–1.3 (1.9) mm × (0.9) 1–1.8 (2.3) mm, (0.4) 0.6–0.8 (1) times longer than wide, usually with two lobate horns, with emargination/horn length (0.1) 0.2–0.5 mm. Fruits glabrous, pruinose-papillose, broadly ovate, (3.6) 3.7–4.4 (4.6) mm × (2.3) 2.4–3.1 mm, 1.3–1.6 (1.8) times longer that wide, styles (0.7) 1.3–1.6-mm long. Seeds ovoid, smooth, yellowish, brownish or greyish, 2.6–2.7 mm × 1.6–1.9 (2) mm, (1.3) 1.4–1.6 (1.7) times longer than wide. Caruncle conical, (0.5) 0.6–0.7 mm × 0.8–1 mm, 0.6–0.8 times longer than wide.
*Distribution:* central and northern Apennine Peninsula to the southern margin of the Alps (Italy), northwest Balkan Peninsula (Croatia: Istria and Kvarner, western Slovenia).
*Habitat:* submediterranean grasslands, scrublands, open forests, and rocky outcrops, mostly over calcareous substrate but also on serpentine.
*Etymology*: We name this species after the Adriatic Sea, on both sides of which it is distributed.

(2) ***Euphorbia hercegovina* Beck** in Glasn. Zemaljsk. Muz. Bosni Hercegovini 32: 95. 1920 ≡ *E. barrelieri* var. *hercegovina* (Beck) Hayek in Repert. Spec. Nov. Regni Veg. Beih. 30: 133. 1924 ≡ *E. barrelieri* subsp. *hercegovina* (Beck) Kuzmanov in Izv. Bot. Inst. (Sofia) 12: 120. 1963 ≡ *Tithymalus barrelieri* subsp. *hercegovinus* (Beck) Soják, Cas. Nár. Mus., Odd. Prír. 140: 170. 1972. — Lectotype (Geltman, 2009, p. 184): [Bosnia and Herzegovina] “Hercegovina, auf dem Leotar,” 7 June 1894, *B[eck] s.n.* (PRC 456036!)^6^.
*Description:* Glabrous and glaucous perennial, (6) 8–17 (22)-cm high, with 1–2-mm thick stems. Terminal rays (4) 5–7 (8), the longest (1.2) 1.9–6.3 (7.9)-cm long, 1–2 times dichotomously branched. Fertile axillary rays (1) 3–6, the longest (1.7) 2.2–6.6 (9.2)-cm long. All leaves with entire margin. Cauline leaves linear-oblanceolate, (0.7) 1.1–2.1 (2.7) cm × (0.2) 0.4–1.1 (1.3) cm, (1.1) 1.6–4.1 (5.4) times longer than wide, widest at (0.5) 0.6–0.8 (0.9) of their length, with cuneate base and obtuse apex. Ray leaves ovate-lanceolate to obovate-lanceolate, (0.8) 0.9–1.4 (1.7) cm × 0.6–1.2 (2) cm, (0.7) 0.8–1.5 (2) times longer than wide, widest at (0.2) 0.3–0.5 (0.6) of their length. Raylet leaves broadly ovate, 0.5–0.9 cm × (0.7) 0.8–1.5 (1.7) cm, (0.4) 0.5–0.7 (0.8) times longer than wide, widest at 0.2–0.3 (0.4) of their length, with cordate base and rounded to obtuse apex. Cyathial involucre campanulate, (1.3) 1.6–2.5 mm × (1.5) 1.7–2.5 (2.6) mm, (0.6) 0.8–1.2 times longer than wide. Cyathial lobes mostly pubescent. Cyathial glands obovate-truncate, (0.6) 0.7–1.4 (1.5) mm × (1) 1.2–1.9 (2.2) mm, 0.4–0.9 (1.1) times longer than wide, usually with two horns, with emargination/horn length of 0.2–0.4 (0.5) mm. Fruits glabrous, pruinose-papillose, broadly ovate, (4.6) 4.7–5.2 mm × (2.8) 2.9–3.7 (3.8) mm, 1.4–1.6 (1.7) times longer than wide; styles, 1.4–1.6-mm long. Seeds ovoid to ellipsoid, smooth, yellowish-brown or grey, 2.5–2.8 mm × 1.6–1.8 (1.9) mm, (1.4) 1.5–1.6 times longer than wide. Caruncle conical, 0.6–0.7 mm × (0.7) 0.8–0.9 (1) mm, 0.7–0.8 (0.9) times longer than wide.
*Distribution:* central Balkan Peninsula (Bosnia and Herzegovina, Croatia, Montenegro).
*Habitat:* on dolomitic substrate in rocky and gravely grasslands, garrigues, and open scrublands, pine forests.

(3) ***Euphorbia japygica* Ten.**, Fl. Napol. 4: 266. 1830 *≡ E. nicaeensis* var. *japygica* (Ten.) Nyman, Consp. Fl. Eur.: 653. 1881 ≡ *E. nicaeensis* subsp. *japygica* (Ten.) Arcang., Comp. Fl. Ital.: 620. 1882 **≡** *E. seguieriana* var. *japygica* (Ten.) Fiori, Fl. Italia 2: 286. 1901 **≡** *Tithymalus nicaeensis* subsp. *japygicus* (Ten.) Soják, Cas. Nár. Mus., Odd. Prír. 140: 174. 1972. — Type: not designated (not in FI, NAP nor RO).
*Note:* According to Fenu et al. (2016), *E. japygica* differs from *E. adriatica* in the fruits being hairy, whereas, in the latter, the fruits are glabrous. The only specimen of the former taxon included in our morphometric study (No. 58), in addition to having hairy fruits, also had larger fruits and longer styles than any measured specimen of *E. adriatica*, but additional morphometric studies are needed to clarify the morphological divergence between both taxa to generate a description for *E. japygica* and clarify its taxonomic status; its treatment as species in this paper should thus be seen as preliminary. It should also be examined whether all populations of this taxon are polyploid and whether the Albanian and some North Macedonian populations treated as *E. nicaeensis* (Barina, 2017) or *E. glareosa* (Micevski, 1998) belong to this species. Also, a type specimen needs to be designated, as there is no original material available in herbaria FI, NAP, and RO, where Tenore’s specimens are deposited. In addition, despite citing “Flora napolitana tav. 232. A” in description of *E. japygica* (Tenore, 1830, p. 266), on the illustration nr. 232, *E. esuloides* Ten. is depicted, and there are no further indications (e.g., in indices of Flora Napolitana) of existence of an illustration of *E. japygica* that could potentially serve as a lectotype.
*Distribution:* southern Apennine Peninsula (Italy: Basilicata, Puglia, doubtful in Campania; Fenu et al., 2016).
*Habitat:* arid grasslands and garrigues up to 1,000 m.

(4) ***Euphorbia macroclada* Boiss**., Diagn. Pl. Orient., sér. 1, 5: 54. 1844 ≡ *Tithymalus macrocladus* (Boiss.) Klotzsch et Garcke in Abh. Königl. Akad. Wiss. Berlin 1859: 97.1860. — Lectotype (Khan, 1964, p. 119): [Turkey], “Denisleh [Denizli] ad collibus argillosis,” June 1842, *Boissier s.n.*, (G-BOIS barcode G00754301 – image!).
= *E. schizoceras* Boiss., Diagn. Pl. Orient., sér. 1, 5: 54. 1844 ≡ *Tithymalus schizoceras* (Boiss.) Klotzsch et Garcke in Abh. Königl. Akad. Wiss. Berlin 1859: 98. 1860 ≡ *E. tinctoria* Boiss. et Huet var. *schizoceras* (Boiss.) Boiss., in DC., Prodr. 15, 2: 166. 1862 — Lectotype (Geltman, 2015, p. 130): “Kurdistan, Berg Gara,” 3 August [1841], *Th. Kotschy570* (G-BOIS barcode G00754277 – image!; isolectotypes: BM 000951553, G00313291, G00390389, G00421116 – image!, K 001080072, LE 01071163, 01071180).
= *E. lorentii* Hochst. in J. A. Lorent, Wanderungen im Morgenland…: 344. 1845. —Type, unknown (not at TUB!). Locality indicated in the protologue: [Syria] “bei Latakia.”
= *E. syspirensis* K. Koch in Linnaea, 21: 727. 1848 ≡ *Tithymalus syspirensis* (K. Koch) Klotzsch et Garcke in Abh. Königl. Akad. Wiss. Berlin 1859: 97. 1860. — Type, unknown. Locality indicated in the protologue: “Im Gaue Sber auf Porphyr und Kieselschiefer, c. 4,000’ hoch.”
= *E. damascena* Boiss., Diagn. Pl. Orient., sér. 1, 12: 113. 1853 ≡ *Tithymalus damascenus* (Boiss.) Klotzsch et Garcke in Abh. Königl. Akad. Wiss. Berlin, 1859: 96. 1860. — Lectotype (Geltman, 2020): “Syria, Damasci collis,” May–July 1846, *E. Boissier* (G-BOISS barcode G00754311 – image!).
= *E. noeana* Boiss. in DC., Prodr. 15, 2: 166. 1862. Pro syn.: “in pl. Noé exs.”
= *E. tinctoria* Boiss. et Huet, in DC., Prodr. 15, 2: 166. 1862. — Lectotype (Geltman, 2006: 164): [Turkey], “Elmali, Gémichem quine dans les ravins,” 9 July 1860, *Bourgeau598*,” (G-BOISS barcode G00754304 – image!; isolectotypes: G00390388, G-DC G00313297 – image!, MPU014638 – image!).
= *E. macroclada* var. *aceras* Hand.-Mazz. in Ann. K. K. Naturhist. Hofmus. 26: 140. 1912. — Lectotype (designated here): [Mesopotanien-Expedition des naturwissenschaftl. Orientvereines in Wien; Turkey], “Kurdistania occidentalis: Taurus Cataonicus. Inter urbem Malatja et vicum Kjachta, in lapidosis et glareosis inter Kory et Furendscha, substrato calcareo, 1,200–1,900 m,” 19 July 1910, *H. F. Handel-Mazzetti2492*. (WU 046588 — image!).
*Description:* Glabrous or pubescent perennial, (18) 29–62 (69)-cm high, with (3) 4–5 (6)-mm thick stems. Terminal rays (3) 5–8 (9), the longest (1.6) 4.1–9.7 (12)-cm long, 1–2 (3) times dichotomously branched. Fertile axillary rays (1) 4–13 (15), the longest (2.7) 4.9–10.7 (12.9)-cm long. All leaves with entire margin. Cauline leaves lanceolate to oblanceolate, (3) 4–6.3 (7.6) × (0.6) 0.7–1.6 (1.9) cm, (2.9) 3.6–6.2 (7) times longer than wide, widest at (0.4) 0.5–0.7 (0.8) of their length, with cuneate base and acute apex. Ray leaves broadly ovate to obovate, (1.1) 1.2–2.6 (3.6) cm × (0.8) 1–1.8 (3) cm, (0.8) 0.9–1.7 (3) times longer than wide, widest at 0.2–0.5 (0.6) of their length. Raylet leaves broadly ovate to reniform, 0.7–1.2 (1.8) cm × 1–1.9 (2.6) cm, (0.5) 0.6–0.8 (1.1) times longer than wide, widest at (0.1) 0.2–0.4 (0.5) of their length, with cordate base and rounded to mucronate apex. Cyathial involucre campanulate, (1.4) 1.8–3.1 (3.5) mm × (2.2) 2.4–3.6 (3.8) mm, (0.4) 0.6–1.2 (1.3) times longer than wide. Cyathial lobes usually densely pubescent. Cyathial glands obovate-truncate, (0.7) 1–2 (2.4) mm × (1.3) 1.5–2.7 (3.3) mm, (0.5) 0.6–0.9 (1.1) times longer than wide, usually with two, often lobate or multifidi, horns, with gland emargination/horn length (0.1) 0.2–0.7 (0.8) mm. Fruits glabrous or pubescent, pruinose-papillose, broadly ovate, (5.9) 6.1–8.4 (8.8) mm × (3.1) 4.1–5.7 (6.2) mm, (1.2) 1.4–1.6 (1.9) times longer than wide, styles (1.3) 1.6–2.5 (2.8)-mm long. Seeds ovoid to ellipsoid, smooth, white, yellow or brown, (2.9) 3.1–4 (4.2) mm × (1.9) 2–2.5 (2.6) mm, (1.2) 1.4–1.9 (2) times longer than wide. Caruncle conical, (0.8) 0.9–1 mm × 1.1–1.2 (1.3) mm, 0.7–0.9 times longer than wide.
2*n* = 18 (Lessani and Chariat-Panahi, 1979; Chariat-Panahi et al., 1982; Fasihi et al., 2016), 36 (Genç and Kültür, 2020).
*Distribution:* Anatolia (Turkey), Armenian Highlands (Armenia), Iranian Plateau (Iran), Levant (Iraq, Israel, Jordan, Lebanon, Syria); Irano-Turanian element.
*Habitat:* stony steppes and scrubland, semideserts, fallow fields, mostly in mountaneous areas.

(5) ***Euphorbia nicaeensis* All.**, Fl. Pedem. 1: 285. 1785 *≡ Galarhoeus nicaeensis* (All.) Haw., Syn. Pl. Succ.: 144. 1812 ≡ *Tithymalus nicaeensis* (All.) Klotzsch and Garcke in Abh. Königl. Akad. Wiss. Berlin, 1859: 89. 1860. ≡ *Esula nicaeensis* (All.) Fourr. in Ann. Soc. Linn. Lyon, n.s., 17: 150. 1869 *≡ Euphorbia seguieriana* var. *nicaeensis* (All.) Fiori in A. Fiori and al., Fl. Italia 2: 286. 1901. – Lectotype (designated here): Herbarium Allioni, *Euphorbia nicaeensis*. (TO, s.n. – image!).
Note: There is only a single specimen of *E. nicaeensis* in the herbarium of Allioni at TO. Despite the fact that there were no locality data stated on the label, we considered the specimen as a part of the original material and selected it here as a lectotype. The locality cited in the protologue is the following: [France], In Comitatu Nicaeensi [Nice] inter Cimie [Cimiez], and la Trinità [La Trinité].
= *E. nicaeensis* var. *aurasiaca* Maire in Bull. Soc. Hist. Nat. Afrique N. 31: 40. 1940. – Lectotype (designated here): B. Balansa, Pl. D’Algerie, 1853 “Lambčse, dans les bois,” 16 July 1853, *Balansa1005* [sub *E. luteola*] (MPU004312 – Image!).
*= E. demnatensis* Coss. ex Batt. in J. A. Battandier and L. C. Trabut, Fl. Algérie, Dicot.: 802. 1890 *≡ E. nicaeensis* var. *demnatensis* (Coss. ex Batt.) Maire in Mém. Soc. Sci. Nat. Maroc 7: 178. 1924. ≡ *E. nicaeensis* subsp. *demnatensis* (Coss. ex Batt..) Breistr. in?. – Lectotype (designated here): Socété dauphinoise, 1883, “Djebel Bouachfal, prov. de Demnat (Maroc),” 3 August 1882, *Ibrahim 4007* (P00540548 – Image!).
= *E. dasycarpa* Coss. ex Batt. in J. A. Battandier and L. C. Trabut, Fl. Algérie, Dicot.: 802. 1890 ≡ *E. nicaeensis* All. var. *dasycarpa* (Coss. ex Batt. and Trab.) Maire in Mém. Soc. Sci. Nat. Maroc 7: 178. 1924. – Lectotype (designated here): “In monte Djebel Afougueur ad austro-occidentem Urbis Maroc, Imperio Maroccano,” 1 June 1876, *Ibrahim* (P05546285 – Image!).
*= E. nicaeensis* var. *hebecarpa* DC. in J. B. A. M. de Lamarck and A. P. de Candolle, Fl. Franç., ed. 3, 5: 363. 1815. – Lectotype (designated here): “Euphorbia myrsinites L., Pyr. or. [ = Pyrénées orientales].” 1814, *J. Coder 226.* (G00313242 – Image!). Note: despite the fact that de Candolle labelled this specimen as “Euphorbia nicaeensis γ” and, in the protologue, γ corresponds to var. *salzmanii*, the collector of the specimen “Coder” corresponds to the indication in the protologue for *E. nicaeensis* var. *hebecarpa*. Moreover, the fruits of the specimen are slightly pilose (“capsulis pilosiusculis”).
= *E. nicaeensis* var. *oleifolia* DC. in J. B. A. M. de Lamarck and A. P. de Candolle, Fl. Franç., ed. 3, 5: 363. 1815. – Lectotype (designated here): “Euphorb. oleafolia Gouan. Castelnau,” 1807, *Dufour s.n.* (G00313234 – Image!).
= *E. nicaeensis* var. *salzmanii* DC. in J. B. A. M. de Lamarck and A. P. de Candolle, Fl. Franç., ed. 3, 5: 363 (1815). – Lectotype (designated here): “Euphorbia nicaeensis var. invollanceol. Gravels,” 1810, *Salzman s.n.* (G00313235 – Image!). Note: despite the fact that de Candolle labelled this specimen as “Euphorbia nicaeensis δ” and, in the protologue, δ corresponds to var. *hebecarpa*, the locality “Gravels” written on the label corresponds to “Grabels prés Montpellier” indicated in the protologue for *E. nicaeensis* var. *salzmanii*. Moreover, Salzman is also indicated as a collector in the protologue, and the fruits of the specimen are glabrous (“capsulis glabris”).
*= E. nicaeensis* subsp. *hispanica* Degen and Hervier in Bull. Acad. Int. Géogr. Bot. 17: 205. 1907. *≡ E. nicaeensis* var. *hispanica* (Degen and Hervier) Cuatrec. in Trab. Mus. Ci. Nat. Barcelona 12: 354. 1929. – Lectotype (designated by Font Garcia et al., 1997): “Barranco del Rio Segura, lieux arides et calcaries, 1,500 m,” July 1906, *Reverchon 1162* (MA-01-00075510 – Image!).
*Description:* Glaborus or pubescent perennial, (5) 15–33 (39)-cm high, with 2–4-mm thick stems. Terminal rays (5) 6–12 (13), the longest (1.2) 2.8–7.1 (8.1)-cm long, 1–2 times dichotomously branched. Fertile axillary rays (1) 2–9 (10), the longest (1.2) 2–7.2 (8.1)-cm long. All leaves with entire margin. Cauline leaves linear-oblanceolate, (1.7) 2.2–3.8 (5) cm × (0.5) 0.6–1 cm, (2.3) 3.1–5.4 (6.5) times longer than wide, widest at 0.6–0.8 of their length, with cuneate base and acute apex. Ray leaves broadly ovate to obovate, (0.7) 1.2–2 (3) cm × (0.5) 0.9–1.5 (1.6) cm, (0.8) 1–2.2 (2.6) times longer than wide, widest at (0.2) 0.3–0.6 (0.7) of their length. Raylet leaves broadly ovate to reniform, (0.5) 0.7–1.2 (1.3) cm × (0.4) 1–1.6 (1.7) cm, (0.4) 0.6–1.1 (1.9) times longer than wide, widest at (0.1) 0.2–0.4 (0.5) of their length, with cordate base and rounded to obtuse apex. Cyathial involucre campanulate, (1.4) 1.7–3 (3.2) mm × (1.3) 1.6–3 (3.3) mm, (0.7) 0.8–1.4 (1.7) times longer than wide. Cyathial lobes are usually pubescent. Cyathial glands obovate-truncate, (0.7) 0.8–1.5 (1.7) mm × (0.9) 1.1–1.9 (2.1) mm, (0.4) 0.6–0.9 (1) times longer than wide, usually with two, often lobate, horns, with emargination/horn length (0.1) 0.2–0.5 (0.6) mm. Fruits glabrous or pubescent, pruinose-papillose, broadly ovate, (4) 4.3–5.7 (6) mm × (2.7) 3.1–4.1 (4.2) mm, 1.3–1.5 (1.6) times longer than wide, styles (0.9) 1.9–2.2-mm long. Seeds, ovoid, smooth, yellowish-brown or grey, 2.7–3.1 (3.3) mm × 1.8–2.1 mm, 1.5–1.7 (1.8) times longer than wide. Caruncle conical, (0.5) 0.6–0.8 mm × (0.8) 0.9–1.1 mm, 0.6–0.8 times longer than wide.
2*n* = 18 (Perry, 1943; Löve, 1978). The chromosome count of 2*n* = 56 by Vilatersana and Bernal (1992) is likely erroneous, as it is their count of 2*n* = 40 for *E. seguieriana* (see Frajman et al., 2019), and it might be due to inappropriate fixation used in the study (R. Vilatersana, written communication to B. Frajman, 2.1.2018).
*Distribution:* northern Algeria and Morocco, Iberian Peninsula (Spain and Portugal) and southern France.
*Habitat:* mountainous areas of the western Mediterranean Basin.

## Data Availability Statement

The datasets presented in this study can be found in online repositories. The names of the repositories and accession numbers can be found in the article/Supplementary Material. The RADseq data are available in the NCBI Short Read Archive (SRA; BioProject ID PRJNA761526, BioSample accessions SRR15817339-SRR15817453) and the ITS sequences in GenBank (GenBank numbers are listed in Supplementary Table 1).

## Author Contributions

BF conceived and designed the study, collected the plant material, coordinated the lab work, performed the data analyses, and wrote substantial parts of the manuscript. VS performed parts of the lab work, morphometric measurements, data analyses, and wrote substantial parts of the manuscript. EZ coordinated the lab work connected to RAD sequences and performed all analyses of RADseq data, wrote corresponding parts of the manuscript, and commented on other parts of the manuscript. All authors contributed to the article and approved the submitted version.

## Conflict of Interest

The authors declare that the research was conducted in the absence of any commercial or financial relationships that could be construed as a potential conflict of interest.

## Publisher’s Note

All claims expressed in this article are solely those of the authors and do not necessarily represent those of their affiliated organizations, or those of the publisher, the editors and the reviewers. Any product that may be evaluated in this article, or claim that may be made by its manufacturer, is not guaranteed or endorsed by the publisher.

## Footnotes

1
http://csf.ac.at/ngs/

2
https://github.com/wtsi-npg/illumina2bam

3
https://www.biostars.org/p/55555/

4
https://osf.io/vqm7r/

5
https://w.jacq.org/W0164201

6
https://prc.jacq.org/PRC456036
