# Supplementary material for: From Western Asia to the Mediterranean Basin: Diversification of the Widespread Euphorbia nicaeensis Alliance (Euphorbiaceae)
Source: Front Plant Sci. 2022 Jun 23;13:815379. doi: 10.3389/fpls.2022.815379 (PMC9262032; doi:10.3389/fpls.2022.815379)
Supplement: Supplementary file 3 [file Data_Sheet_1.PDF]

Supplementary Material

From western Asia to the Mediterranean Basin: diversification of the widespread *Euphorbia nicaeensis* alliance (Euphorbiaceae)

VALENTINA STOJILKOVIČ[1,2], ELIŠKA ZÁVESKÁ[1,3], BOŽO FRAJMAN[1*]

[1]*Department of Botany, University of Innsbruck, Sternwartestraße 15, 6020 Innsbruck, Austria*

[2]*Department of Biology, Biotechnical Faculty, University of Ljubljana, Večna pot 111, 1000 Ljubljana, Slovenia*

[3]*Institute of Botany of the Czech Academy of Sciences, Zámek 1, 252 43 Průhonice, Czech Republic,*

[*]Corresponding author. E-mail: bozo.frajman@uibk.ac.at

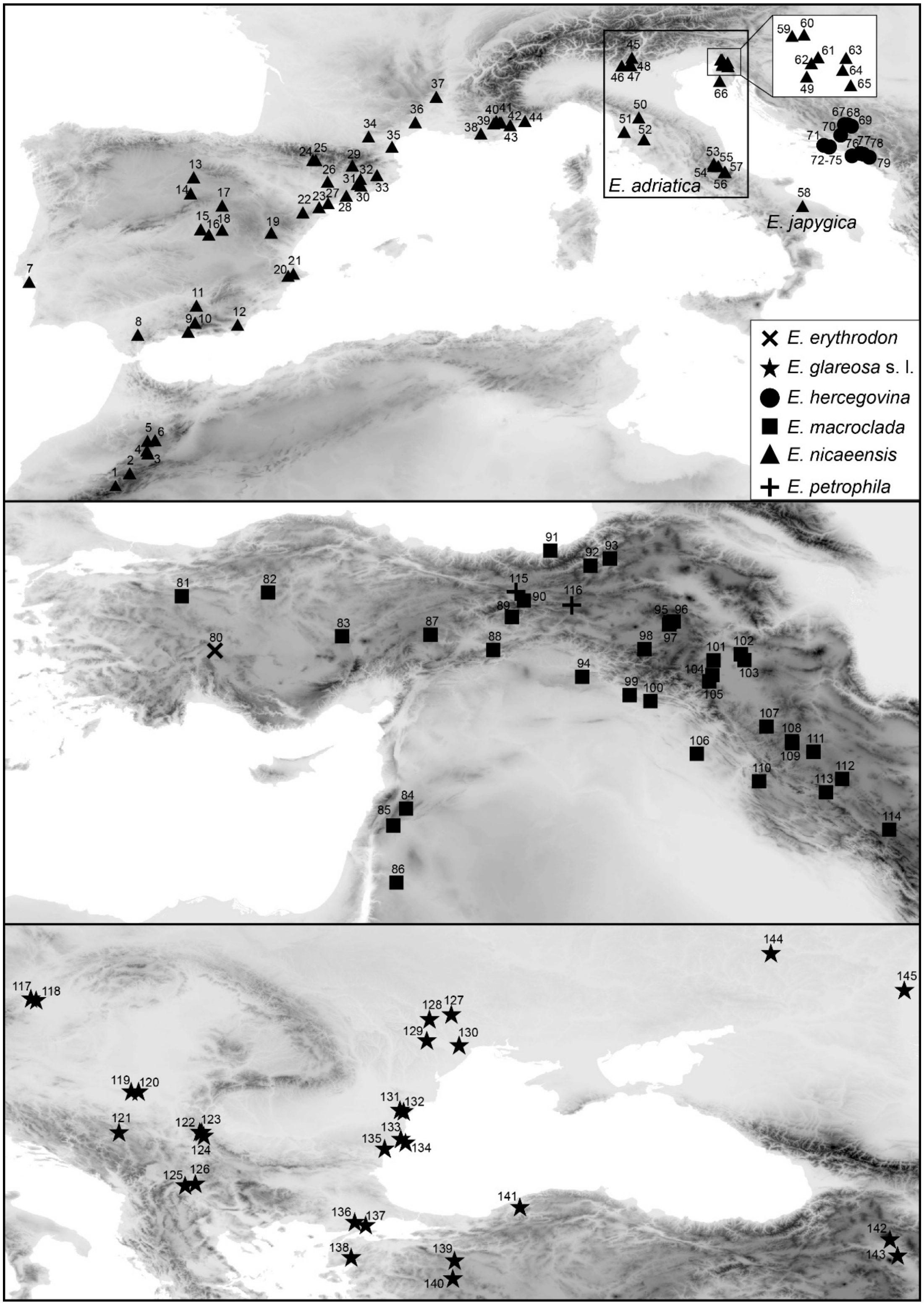

Supplementary Figure 1. Distribution of the populations of the *Euphorbia nicaeensis* alliance used in this study. Population numbers correspond to Supplementary Table 1. Populations 45 to 57 and 59 to 66 that previously belonged to *E. nicaeensis* and are now treated as *E. adriatica* are indicated, as well as the population 58, which is now treated as *E. japygica*.

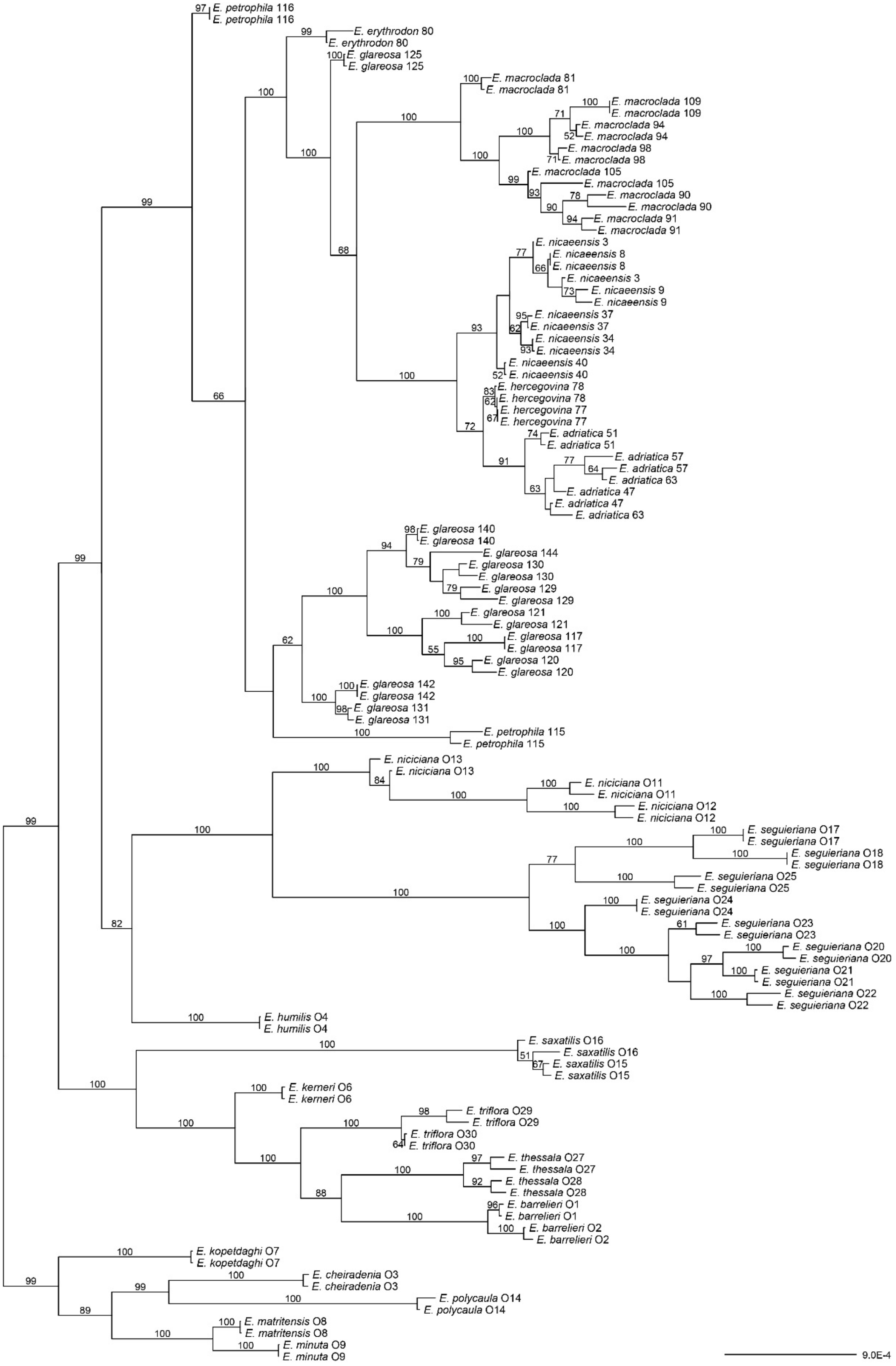

Supplementary Figure 2. Phylogenetic relationships within the *Euphorbia nicaeensis* alliance and between this alliance and its closest relatives within *E.* sect. *Pithyusa* as inferred by maximum likelihood analysis of RADseq loci with indicated bootstrap values above 50%.

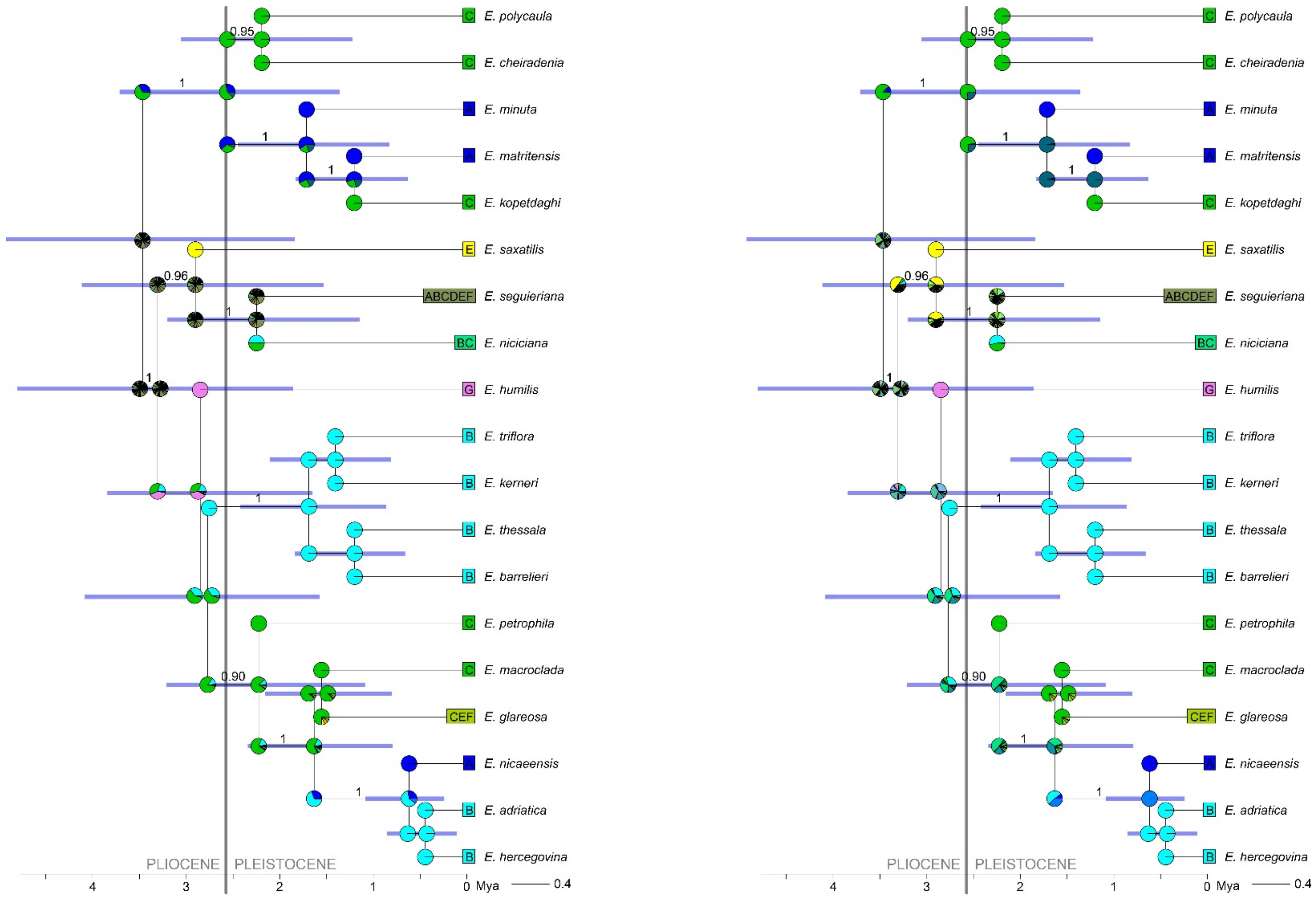

Supplementary Figure 3. Time-calibrated species tree based on RADseq data inferred with SNAPP. Numbers above branches are posterior probabilities > 0.80 and the horizontal bars correspond to 95% highest posterior densities (HPD) of the age estimates. The vertical grey bar shows the boundary between the Pliocene and the Pleistocene. Pie charts at each node show the marginal probabilities of alternative ancestral ranges obtained from the BioGeoBEARS analysis under the DEC + J (left) and DEC (right) models. The colours and the letter codes for distribution areas at present indicated at the terminals correspond to Fig. 2. Black segments in pie charts represent ancestral ranges with a probability < 10%.

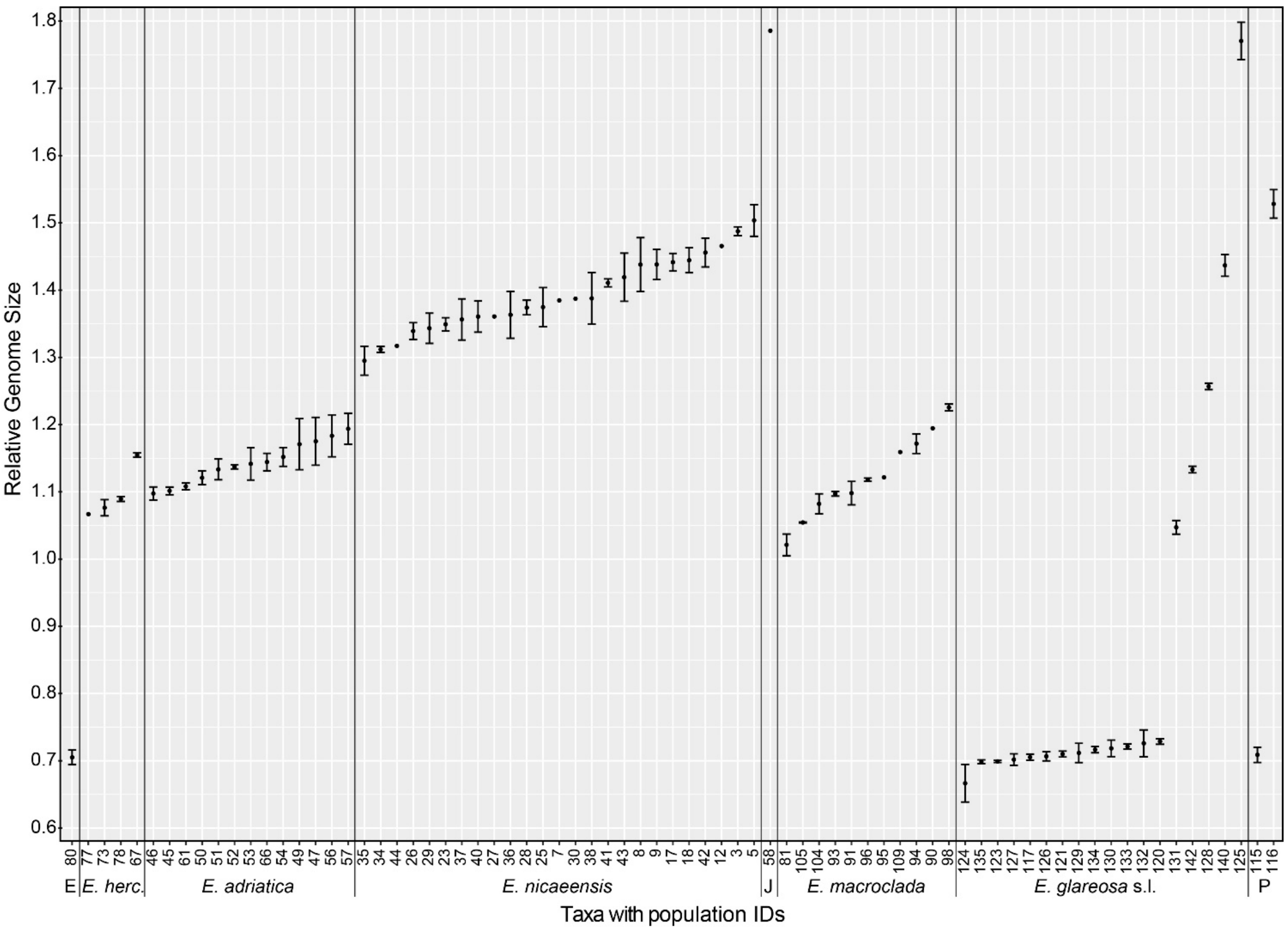

Supplementary Figure 4. Relative genome size (RGS) variation in the *Euphorbia nicaeensis* alliance. Shown are population mean values (dot) and standard deviation (line). Population numbers correspond to Supplementary Figure 1 and Supplementary Table 1, where it is also listed how many individuals per population were analyzed. E, *E. erythrodon*; *E. herc.*, *E. hercegovina*; J, *E. japygica*; P, *E. petrophila*.

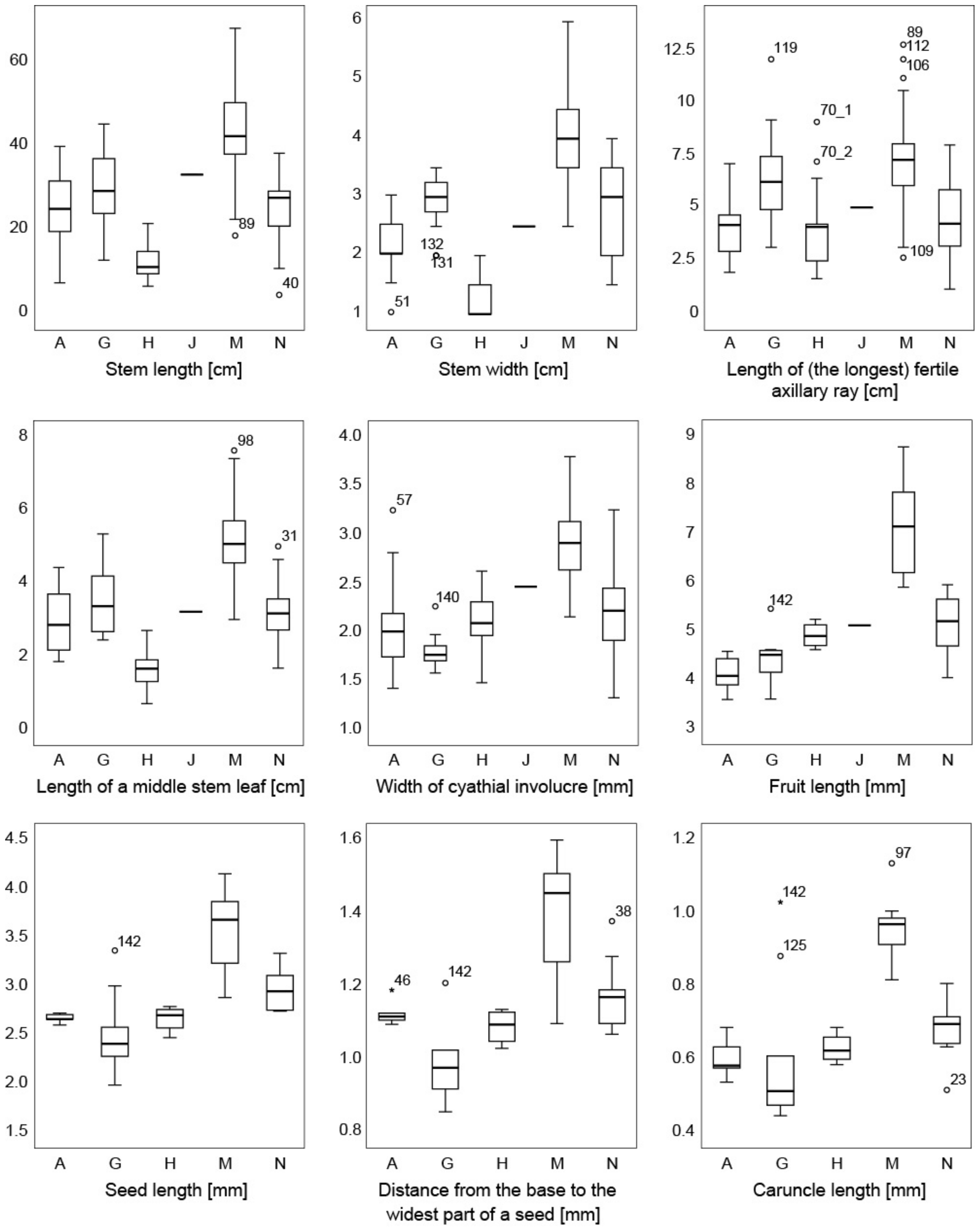

Supplementary Figure 5. Boxplot diagrams of morphological characters discriminating best between the studied taxa of the *Euphorbia nicaeensis* alliance. Boxes define 25 and 75 percentiles; lines indicate medians; whiskers span the 2.5 to 97.5 percentiles, and circles and stars indicate outliers. A, *E. adriatica*; G, *E. glareosa*; H, *E. hercegovina*; J, *E. japygica*; M, *E. macroclada*; N, *E. nicaeensis*.

Supplementary Table 3. Comparison of six biogeographic models used in a common likelihood framework to estimate ancestral areas in the evolution of *E. barrelieri-nicaeensis-seguieriana* clade with the program BioGeoBEARS. The six models differ in a set of parameters that are allowed to vary freely, i.e. range expansion (*d*), range contraction (*e*) and founder-event speciation (*j*). Log likelihood (lnL) and Akaike Information Criterion (AIC) scores are presented. The results for the model BAYAREALIKE + J could not be obtained.

**Model**	**d**	**e**	**j**	**lnL**	**No. of parameters**	**AIC**
**DEC+J**	**0.022**	**1.00E-12**	**0.059**	**-44.24**	**3**	**94.48**
DIVALIKE+J	0.037	1.00E-12	0.044	-45.88	3	97.76
DEC	0.037	1.00E-12	0	-48.29	2	100.59
DIVALIKE	0.052	1.00E-12	0	-48.65	2	101.31
BAYAREALIKE	0.034	3.76E-01	0	-55.96	2	115.93
BAYAREALIKE+J	NA	NA	NA	NA	NA	NA
